# Transcriptome differences between Cry1Ab resistant and susceptible strains of Asian corn borer

**DOI:** 10.1186/s12864-015-1362-2

**Published:** 2015-03-12

**Authors:** Li-Na Xu, Yue-Qin Wang, Zhen-Ying Wang, Ben-Jin Hu, Ying-Hui Ling, Kang-Lai He

**Affiliations:** Institute of Plant Protection and Agro-Products Safety, Anhui Academy of Agricultural Sciences, Hefei, Anhui 230031 China; The State Key Laboratory for Biology of Plant Diseases and Insect Pests, Institute of Plant Protection, Chinese Academy of Agricultural Sciences, Beijing, 100193 China; College of Animal Science and Technology, Anhui Agricultural University, Hefei, Anhui 230036 China

**Keywords:** *Ostrinia furnacalis*, Cry1Ab, Transcriptome differences, Resistance, Strains

## Abstract

**Background:**

Asian corn borer (ACB), *Ostrinia furnacalis* (Guenée), is the major insect pest of maize in China and countries of East and Southeast Asia, the Pacific and Australasia. ACB can develop strong resistance to the transgenic Bt maize expressing Cry1Ab, the most widely commercialized Bt maize worldwide. However, the molecular basis for the resistance mechanisms of ACB to Cry1Ab remained unclear. Two biological replicates of the transcriptome of Bt susceptible (ACB-BtS) and Cry1Ab resistant (ACB-AbR) strains of ACB were sequenced using Solexa/Illumina RNA-Seq technology to identify Cry1Ab resistance-relevant genes.

**Results:**

The numbers of unigenes for two biological replications were 63,032 and 53,710 for ACB-BtS and 57,770 and 54,468 for ACB-AbR. There were 35,723 annotated unigenes from ACB reads found by BLAST searching NCBI non-redundant, NCBI non-redundant nucleotide, Swiss-prot protein, Kyoto Encyclopedia of Genes and Genomes, Cluster of Orthologous Groups of proteins, and Gene Ontology databases. Based on the *NOISeq* method, 3,793 unigenes were judged to be differentially expressed between ACB-BtS and ACB-AbR. Cry1Ab resistance appeared to be associated with change in the transcription level of enzymes involved in growth regulation, detoxification and metabolic/catabolic process. Among previously described Bt toxin receptors, the differentially expressed unigenes associated with aminopeptidase N and chymotrypsin/trypsin were up-regulated in ACB-AbR. Whereas, other putative Cry receptors, cadherin-like protein, alkaline phosphatase, glycolipid, actin, V-type proton ATPase vatalytic, heat shock protein, were under-transcripted. Finally, GPI-anchor biosynthesis was found to be involved in the significantly enriched pathway, and all genes mapped to the pathway were substantially down-regulated in ACB-AbR.

**Conclusion:**

To our knowledge, this is the first comparative transcriptome study to discover candidate genes involved in ACB Bt resistance. This study identified differentially expressed unigenes related to general Bt resistance in ACB. The assembled, annotated transcriptomes provides a valuable genomic resource for further understanding of the molecular basis of ACB Bt resistance mechanisms.

**Electronic supplementary material:**

The online version of this article (doi:10.1186/s12864-015-1362-2) contains supplementary material, which is available to authorized users.

## Backgound

Asian corn borer (ACB), *Ostrinia furnacalis* (Guenée), is the major insect pest of maize in China and countries of East and Southeast Asia, such as Japan, Korea, Thailand, Philippines, Indonesia, Malaysia, as well as the Pacific and Australasia [1]. ACB feeds on the stems, leaves and ears, and can cause yield losses of 20-80% [2]. Transgenic Bt maize, such as those expressing Cry1Ab, Cry1Ac and Cry1Ie, can offer season-long protection against ACB [3-5]. However, the future of Bt maize is threatened by evolution of target insect resistance. Already one ACB strain has developed strong resistance to Cry1Ab, and readily consumed Cry1Ab-expressing maize silks [6]. In addition, resistance to Cry1Ac, Cry1Ie and Cry1F in ACB has been generated by the laboratory selection [7,8]. Understanding how ACB becomes resistant to Bt toxins is needed to develop measures to counter this process.

There are two different hypotheses for Cry toxin action, one dependant on pore formation and the other on signal transduction [9,10]. The first steps in both models are similar: the toxin crystals are ingested by the larvae and solubilized in the gut to pro-toxins, which are cleaved by midgut proteases to give rise to a 60-kDa activated toxin [11]. The activated toxin is able to bind to a cadherin-like receptor that is located in the microvilli of the midgut cells [12]. The pore-formation model proposes that interaction with cadherin-like protein facilitates further proteolytic cleavage [13], resulting in the oligomerization of the toxin. The toxin oligomer then binds to secondary receptors, which are proteins anchored to the membrane, by a glycosylphosphatidylinositol (GPI)-anchor, such as aminopeptidase N (APN) in *Manduca sexta* or alkaline phosphatase (ALP) in *Heliothis virescens* [11,14,15]. In a final step, the toxin oligomer inserts into lipid raft membranes, where it forms pores and subsequently causes cells to burst, resulting in the death of the larva [11,16,17]. By contrast, in the signal-transduction model, the binding of Cry1A to cadherin-like is assumed to trigger a cascade pathway involving the stimulation of a G protein and adenylate cyclase to increase cAMP, resulting in the activation of protein kinase A, which in turn leads to oncotic cell death [18]. Recent studies in various target insect have found some novel putative Bt resistant genes, such as ATP-binding cassette (ABC) transporters [19]. A mutation in a class of ABC transporters was proposed to be associated with Bt resistance in *H. virescens* [20] and mutations in the orthologous ABC transporters (ABCC2) were reported to be associated with Bt resistance in *Trichoplusia ni* and *Plutella xylostella* [21]. Besides, Atsumi et al. provided evidence that Bt resistance was caused by a mutation in an orthologous ABC transporter in *Bombyx mori* by introducing a Bt-susceptible allele into a resistant silkworm using transgenesis [22]. However, Bt resistance is not fully explained by these findings.

Mutations in sequence and mRNA expression of four APN genes between ACB-AbR and ACB-BtS have been identified [23]. Also, V-type ATPase catalytic subunit A and heat shock 70 kDa proteins were identified as the novel candidate Bt toxins receptors in ACB using a proteomic approach [24]. However, the Bt resistance mechanism in ACB remains unclear, and we postulate that resistance to Bt toxins is a complex process involving an array of genetic and metabolic factors.

Gene expression analysis is widely used to reveal regulatory mechanisms that control cellular processes in animal, plants and microbes. In particular, recently developed Solexa/Illumina RNA-Seq and digital gene expression based next generation sequencing technology have substantially changed the way resistance-relevant genes in insects are identified because these methods facilitate investigation of the functional complexity of transcriptome [25,26]. RNA-Seq extends the possibilities of transcriptome studies to the analysis of previously unidentified genes and splice variants. Moreover, RNA-Seq offers an unlimited dynamic quantification range with reduced variability. These advantages, coupled with the declining cost of sequencing, make RNA-Seq an increasingly attractive method for whole-genome expression studies in many biological systems, including species with unsequenced genomes [27].

In this study, a global transcriptome-based analysis of ACB in response to Cry1Ab toxin was examined using bioinformatics techniques coupled with high throughput RNA-Seq. An RNA-Seq transcriptome dataset was obtained from the mixture of 1–5 instar ACB larvae, a set of high-quality ACB gene structures was delineated and functionally annotated, and the transcriptome-level response of ACB larvae to Cry1Ab was analyzed. The differentially expressed transcripts were further validated by quantitative real-time PCR (RT-qPCR) analysis. This study, together with other genomic research, provided a transcriptomic basis for the mechanistic study of Bt resistance in ACB.

## Results

### Illumina sequencing analysis and *de novo* assembly

On an overview of gene expression of ACB, after cleaning and quality checks about 51.5 and 51.8 million reads of 90 bp were obtained from the two replicates of ACB-AbR and 52.95 and 54.67 million from ACB-BtS (Accession No: SRP046207). The clean reads were assembled into 102,236 and 91,311 contigs with mean lengths of 348 and 332 nt for ACB-BtS and 88,634 and 84,209 contigs both with a mean length of 366 nt for ACB-AbR (Table 1). Using paired-end joining and gap-filling, these contigs were further assembled into 63,032 and 53,710 ACB-BtS-unigenes with mean lengths of 607 nt and 580 nt for ACB-BtS and 57,770, and 54,468 ACB-AbR-unigenes with mean lengths of 629 nt and 613 nt for ACB-AbR. The size distribution of these contigs and unigenes were given in Additional file 1: Figure S1 and Additional file 2: Figure S2.

**Table 1:** **Summary of reads in Cry1Ab susceptible strain (ACB-BtS) and resistant strain (ACB-AbR) of** ***Ostrinia furnacalis*** **transcriptomes**

		**ACB-BtS-1**	**ACB-BtS-2**	**ACB-AbR-1**	**ACB-AbR-2**
Total clean nucleotides (nt)		4,631,663,700	4,662,124,200	4,765,847,040	4,920,077,340
Total clean reads		51,462,903	51,801,380	52,953,856	54,667,526
GC percentage (%)		49.50	50.04	48.80	49.22
Total number of contigs		102,236	91,311	88,634	84,209
Mean length of contigs (nt)		348	332	366	607
Total number of unigene		63,032	53,710	57,770	54,468
Mean length of unigene (nt)		607	580	629	613
Distinct clusters		14,628	11,426	12,912	11,397
Distinct singletons		48,350	42,284	44,858	43,071
SNP type	A - G	36,334	34,649	25,361	23,984
	C - T	36,868	35,104	25,385	24,441
	A - C	9,884	8,980	7,092	6,778
	A - T	13,589	12,324	9,887	9,283
	C - G	8,141	7,147	5,797	5,515
	G - T	9,676	8,866	6,855	6,464

### Annotation of assembled unigenes

A total of 61,622 unigenes were detected from the four ACB libraries, among them, 35,723 unique sequences were annotated based on blastx alignment (E-value < 0.00001) searches of six public databases: NCBI non-redundant (NR), NCBI non-redundant nucleotide (NT), Swiss-prot protein, Kyoto Encyclopedia of Genes and Genomes (KEGG), Cluster of Orthologous Groups of proteins (COG), and Gene Ontology (GO) databases (Additional file 3: Table S1). Of these, 30,264 unique sequences were annotated by reference to the NR database. Based on the NR annotations, 45.3% of the annotated sequences had very strong homology (E-value < 10^−60^), and 19.7% showed strong homology (10^−60^ < E-value < 10^−30^) and an additional 35.1% showed homology (10^−30^ < E-value < 10^−5^) to known insect sequences. The similarity distribution was comparable with 26.9% of the sequences having similarities higher than 80%, while 73.1% of the matches had similarities of 16-80%. With respect to species, 60.7% of the unique sequences had highest matches to sequences from *Danaus plexippus*, with additional matches to *B. mori* (7.2%), *Tribolium castaneum* (3.7%), *Papilio xuthus* (3.3%), *Papilio polytes* (1.1%), *Acyrthosiphon pisum* (1.0%) and *Ostrinia nubilalis* (0.8%) (Additional file 4: Figure S3).

**Figure 1:**
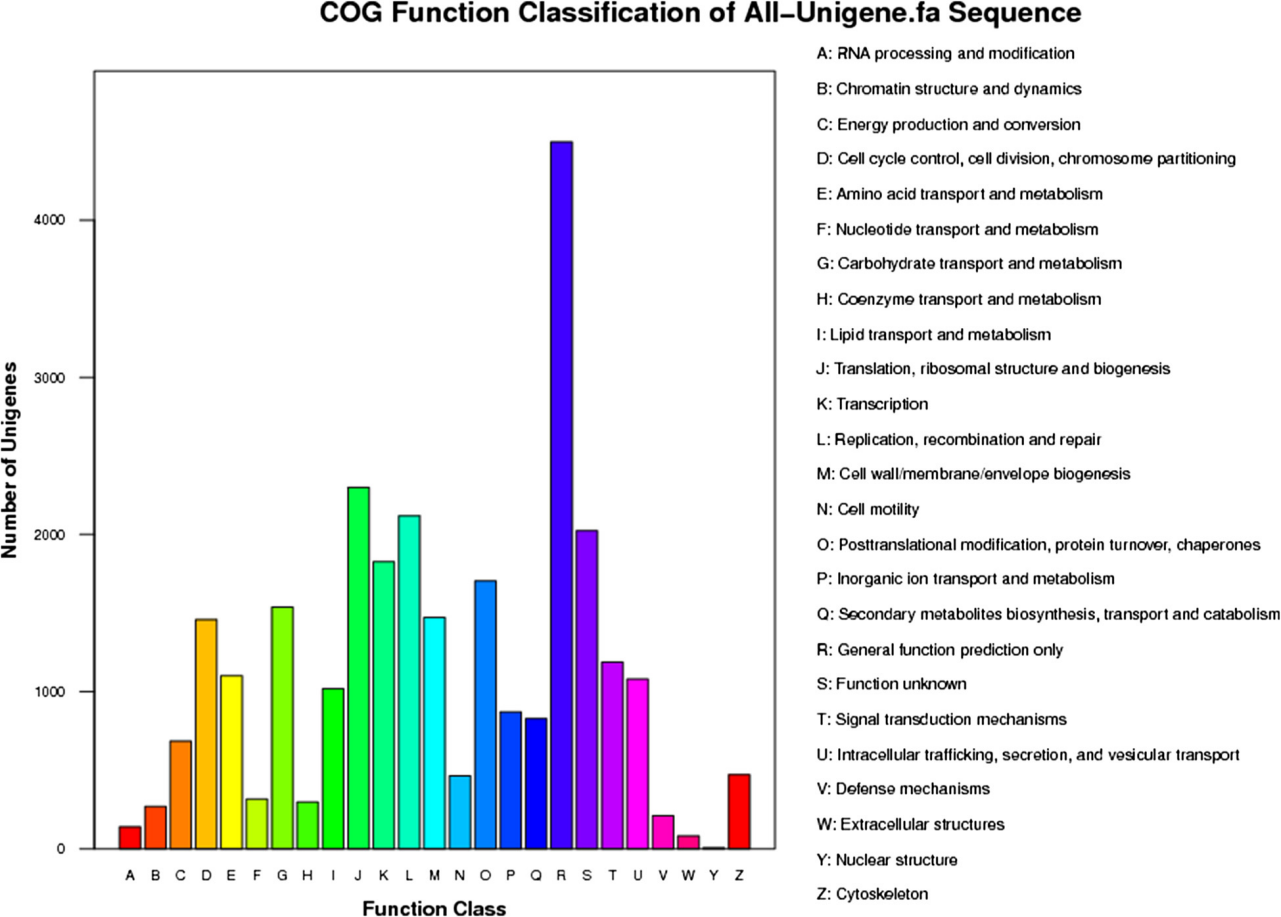
**Histogram of Clusters of Orthologous Groups (COG) classification.** 27952 unigenes were assigned to 25 categories in the COG classification. The right legend shows a description of the 25 function categories.

To further examine the integrity and effectiveness of the annotation process, the unigenes (with NR matches) number with COG classification was calculated. By this means 27,952 unigenes (Additional file 5: Table S2) were identified with a COG classification. Among the 25 COG categories, the cluster of “General function prediction” occupied the highest number (4,499, 16.10%), followed by “Translation, ribosomal structure and biogenesis” (2,300, 8.22%) and “Replication, recombination and repair” (2,120, 7.58%). The categories of “RNA procession and modification” (137, 0.49%), “Extracellular structures” (81, 0.29%) and “Nuclear structure” (5, 0.02%) had the fewest matching genes (Figure 1).

**Figure 2:**
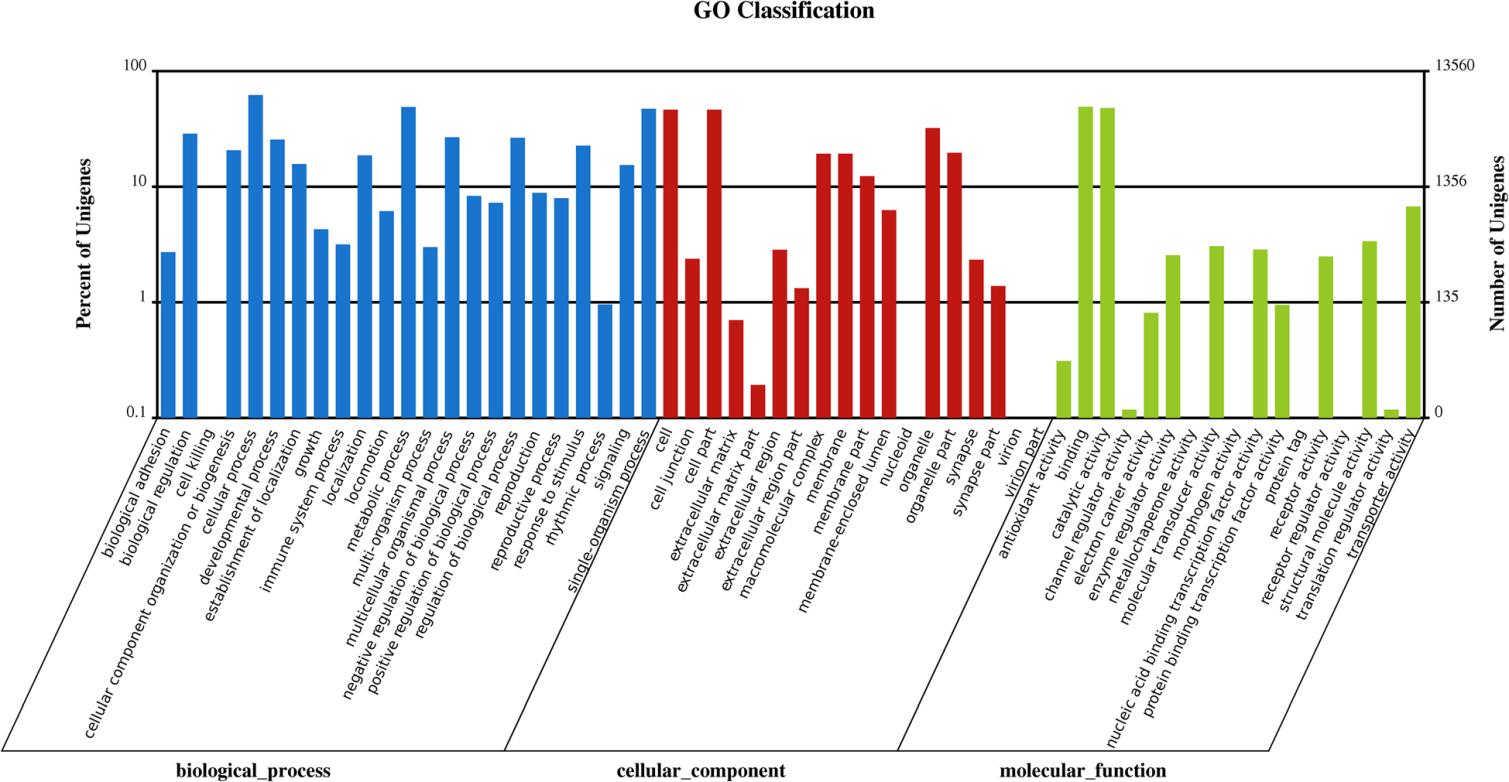
**Histogram of Gene Ontology classification.** Go categories, shown in the x-axis, are grouped into three main ontologies: biological process, cellular component and molecular function. The right y-axis indicates the number of genes in each category, while the left y-axis indicates the percentage of total genes in that category. The “All-unigene” indicated that the unigenes were those assembled from reads from the four samples of *Ostrinia furnacalis*.

GO and KEGG assignments were used to classify the functions of the predicted ACB unigenes. Based on homologous genes, 13,560 sequences (Additional file 6: Table S3) from all unigenes of four ACB libraries were categorized into 58 GO terms consisting of three domains: biological process, cellular component and molecular function (Figure 2). Most were categorized in “cellular process”, “metabolic process”, “binding”, “single-organism process” and “catalytic activity”. A high percentage of genes were also assigned to “cell”, “cell part”, “organelle”, “biological regulation”, and “multicellular organismal process”, and some to “regulation of biological process”, “developmental process”, “response to stimulus”, “cellular component organization or biogenesis”, “macromolecular complex” and “membrane”. However, no genes were assigned to “cell killing”, “nucleoid”, “virion”, “virion part”, “metallochaperone activity”, “morphogen activity”, “protein tag” or “receptor regulator activity” (Figure 2).

There were 20,144 unigenes from all the unigenes of four ACB libraries that mapped into 258 KEGG pathways (Additional file 7: Table S4). The maps with highest unigene representation were metabolic pathways (ko01100; 3514 unigenes, 17.4%), followed by biosynthesis of secondary RNA transport (ko03013; 780 unigenes, 3.9%), purine metabolism (ko00230; 747 unigenes, 3.7%), and spliceosome (ko03040; 735 unigenes, 3.7%).

Totals of 114,492, 107,070, 80,377, and 76,465 SPNs, in which transition (A-G, C-T) accounted for 63.93%, 65.15%, 63.13%, and 63.33%, were predicted in ACB-BtS-1, ACB-BtS-2, ACB-AbR-1, and ACB-AbR-2 through SOAPsnp software (Table 1). A total of 4355 SNPs, which existed on a certain site of four ACB samples but the types were not all the same, were detected in the present study. Meanwhile, 2360 genes were affected by the common SNPs in the four samples (Additional file 8: Table S5). Among them, 18 unigenes annotated to putative Bt toxin receptors were affected by a total of 34 SNPs, which presented the same genotype in the two biological replications of each phenotype, but different between ACB-BtS and ACB-AbR. This included 23 non-synonymous SNPs affecting the protein sequence of 2 cadherin-like proteins, 10 APNs and 1 ALP (Table 2).

**Table 2:** Non-synonymous changes in putative Cry toxin receptors*

**Receptor**	**Gene ID**	**Position cDNA**	**Reference**	**ACB-BtS-1**		**ACB-BtS-2**		**ACB-AbR-1**		**ACB-AbR-2**		**AA change**
				**Base**	**Number**	**Base**	**Number**	**Base**	**Number**	**Base**	**Number**	
APN1	Unigene4279	1849	G	G	231	G	164	A	252	A	248	A > T
APN1	CL3709.Contig2	824	G	G	150	G	231	A	218	A	255	C > Y
	CL3709.Contig2	1010	A	A	108	A	85	G	255	G	255	N > S
	CL3709.Contig2	1034	T	T	116	T	99	G	254	G	255	V > G
	CL3709.Contig2	1124	A	A	31	A	31	G	34	G	29	D > G
APN1	CL3709.Contig5	774	T	T	26	T	17	C	110	C	227	G > G
APN1	CL3709.Contig8	171	A	G	11	G	8	A	11	A	27	S > S
APN2	Unigene9047	479	G	A	85	A	45	G	129	G	145	C > Y
	Unigene9047	650	G	A	35	A	25	G	81	G	161	R > P
	Unigene9047	827	G	C	26	C	13	G	77	G	178	W > S
APN3	CL1804.Contig3	2998	T	C	33	C	23	T	76	T	30	S > P
APN3	CL6793.Contig1	754	A	A	12	A	16	G	12	G	25	N > D
	CL6793.Contig1	775	G	G	19	G	18	A	20	A	22	A > T
	CL6793.Contig1	1174	G	G	23	G	22	A	22	A	22	E > K
APN3	CL9145.Contig1	1303	T	T	41	T	21	C	37	C	81	S > P
APN3	Unigene11364	688	G	G	20	G	24	A	45	A	67	G > S
APN3	Unigene431	1249	G	G	16	G	15	A	34	A	37	V > I
	Unigene431	1351	A	A	23	A	23	C	42	C	37	N > H
APN3	Unigene5966	134	T	T	25	T	8	A	36	A	41	F > Y
APN7	Unigene12395	521	T	T	41	T	20	C	41	C	64	I > T
	Unigene12395	805	G	G	92	G	47	A	131	A	147	V > I
	Unigene12395	1268	C	C	66	C	24	T	103	T	104	P > L
	Unigene12395	1381	T	T	229	T	137	C	185	C	255	Q > Q
APN12	Unigene3586	1146	T	T	22	T	29	G	8	G	8	S > S
APN7	Unigene887	243	T	C	129	C	68	T	74	T	87	V > V
	Unigene887	252	T	C	117	C	61	T	78	T	88	D > D
	Unigene887	261	C	T	117	T	62	C	83	C	93	N > N
	Unigene887	270	T	C	108	C	62	T	82	T	93	C > C
Cadherin-like protein	CL8807.Contig8	2798	C	T	56	T	33	C	62	C	54	P > P
	CL8807.Contig8	3110	G	T	149	T	68	G	127	G	76	V > G
Cadherin-like protein	Unigene3408	1368	T	A	166	A	202	T	171	T	159	G > V
	Unigene3408	1934	A	A	161	A	227	T	254	T	254	R > R
ALP	CL2676.Contig2	474	A	G	8	G	4	A	31	A	25	N > I
ALP	CL944.Contig2	2955	C	T	43	T	33	C	17	C	21	F > L

*Position: the position of SNP existed in a certain Unigene; Base: best nucleotide covering the position; Number: number of the best nucleotide on the certain position; AA change: amino acid change.

### Differential expression analysis and RT-qPCR validation in ACB-AbR and ACB-BtS

**Figure 3:**
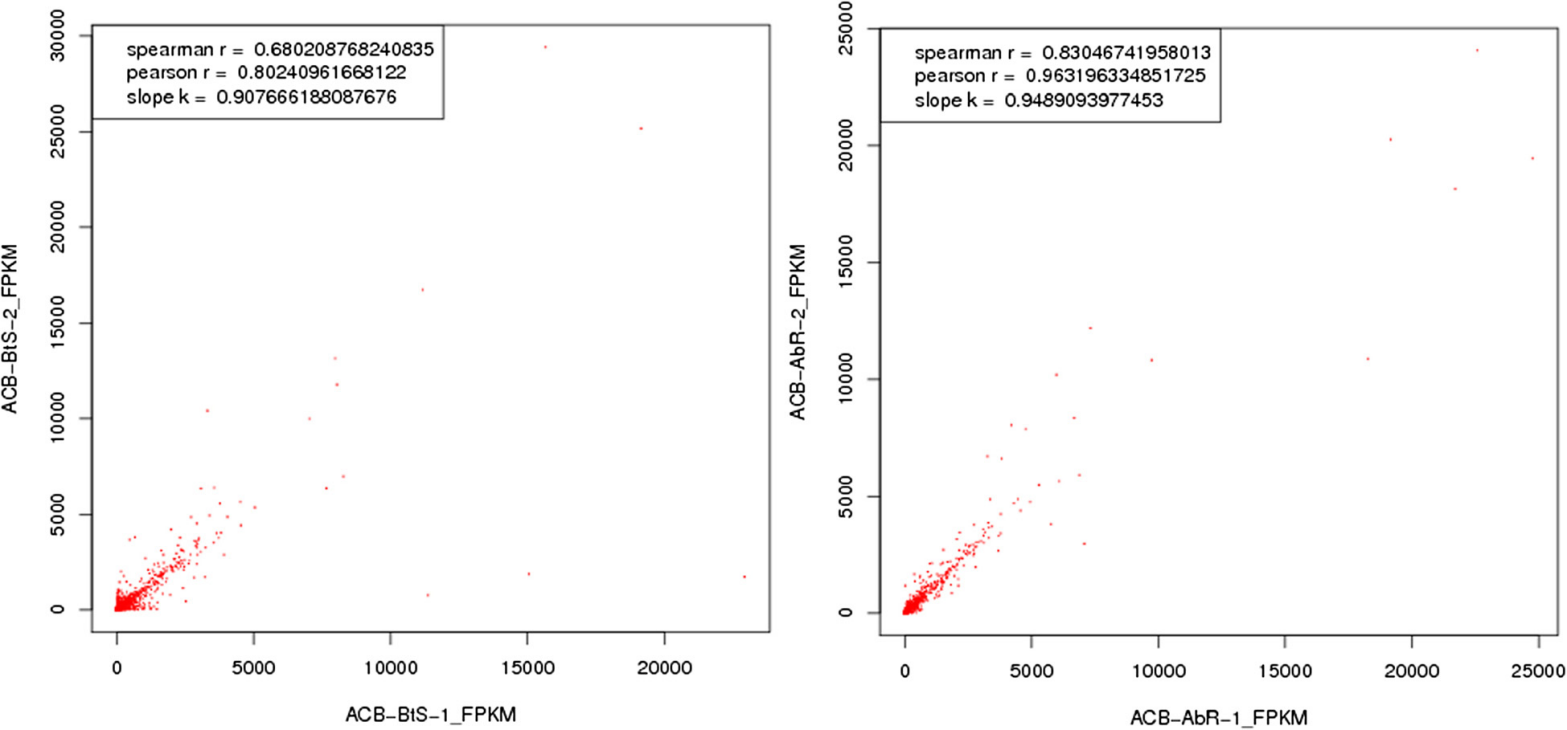
**Pearson correlation analysis of replicates from Cry1Ab susceptible (ACB-BtS) and resistant (ACB-AbR) strains of** ***Ostrinia furnacalis.*** The left is analyze of ACB-BtS, and the right one is ACB-AbR.

**Figure 4:**
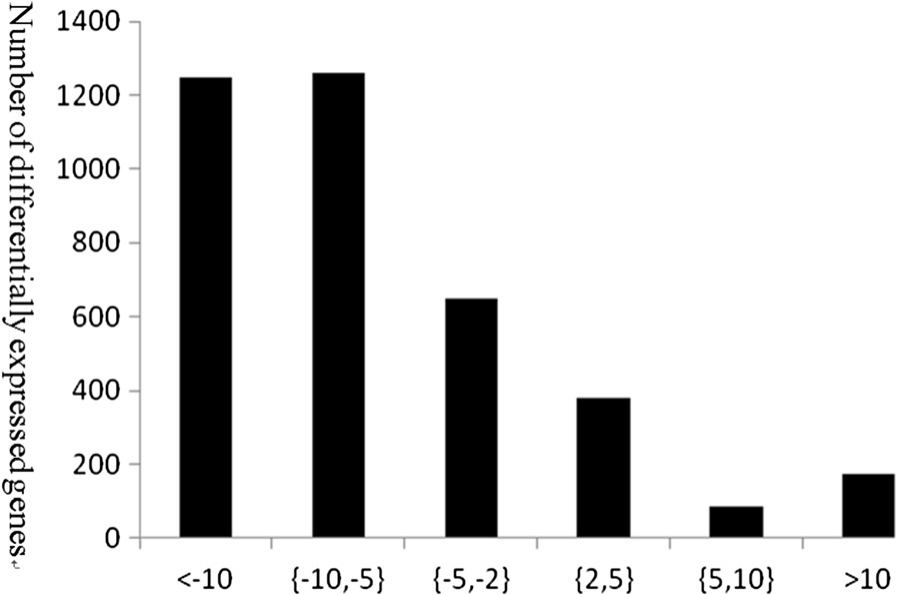
**Change distribution for unigenes differentially expressed between Cry1Ab susceptible (ACB-BtS) and resistant (ACB-AbR) strains of** ***Ostrinia furnacalis.***

The Pearson coefficient (r = 0.96) of all unigenes in ACB-AbR-1 and ACB-AbR-2 indicated an acceptable reproducibility, however, for replicates from ACB-BtS the coefficient (r = 0.80) was less satisfactory (Figure 3). Therefore, the significance of gene expression difference between ACB-BtS and ACB-AbR was assessed using the *NOISeq* method with the option: *Q*-value ≥ 0.8, relative change ≥ 2, based on the clean reads of four ACB libraries. ACB-AbR had 636 up-regulated and 3157 down-regulated unigenes when compared to the gene expression of ACB-BtS. Among all differentially expressed unigenes (DEUs) in ACB-AbR, 173 unigenes were up-regulated and 1247 down-regulated more than 10-times (Figure 4).

Among the DEUs, 12 (0.31%) were found to encode detoxification enzymes including glutathione S-transferase (GST), cytochrome P450 (P450) and carboxylesterase (CaE). The unigenes associated with GST and P450 were down-regulated in ACB-AbR (2.94 to 7.94 times), whereas, the unigenes annotated to CaE were up-regulated. Meanwhile, 10 DEUs (0.29%) were annotated to chymotrypsin or trypsin, potentially involved in Cry protoxin activationwere over-transcribed in ACB-AbR (2.50 to 4.58 times).

In the case of resistance candidate Bt receptor genes, most specific genes such as cadherin-like protein, glycolipid, actin, V-type proton ATPase vatalytic, heat shock protein were under-transcribed in ACB-AbR, however, the DEUs annotated to APNs were up-regulated (Table 3). In addition, no DEU was annotated to ALP for ACB-AbR. Only one unigene (CL7354.Contig2) associated to the ALP pathway (ko01113) was under-transcripted in ACB-AbR.

**Table 3:** **The differentially expressed of candidate Bt receptor genes between the Cry1Ab susceptible strain (ACB-BtS) and Cry1Ab resistant strain (ACB-AbR) of** ***Ostrinia furnacalis*** *

**Unigene ID**	**Log2Ratio**	**Gene length**	**Annotation**	**ID in database**	***Q*** **-value**
CL951.Contig2	−3.02252	2495	glutathione S-transferase 8 [*Bombyx mori*]	NP001108463.1	0.834605
Unigene20656	−6.32071	451	glutathione S-transferase-like [*Papilio xuthus*]	BAM18639.1	0.800144
CL686.Contig1	−4.13653	1770	cytochrome P450 4c3 [*P. xuthus*]	BAM19419.1	0.858289
CL2256.Contig1	−6.78223	1325	cytochrome P450 [*P. xuthus*]	BAD99563.1	0.87369
CL686.Contig7	−2.94602	1753	cytochrome P450 4c3 [*P. xuthus*]	BAM19419.1	0.833112
Unigene30330	−4.13052	233	cytochrome P450 [*B. mori*]	BAM73826.1	0.808007
Unigene32262	−5.16389	487	cytochrome P450, partial [*B. mori*]	BAM73834.1	0.818636
Unigene17691	−7.94213	1472	cytochrome P450 CYP6CT1 [*Danaus plexippus*]	EHJ78442.1	0.821273
Unigene21220	−3.25656	1946	cytochrome P450 [*B. mori*]	BAM73795.1	0.850597
CL686.Contig4	−4.54016	1647	cytochrome P450 4c3 [*P. xuthus*]	BAM19419.1	0.860983
CL6671.Contig1	5.177787	2090	carboxylesterase [*Loxostege sticticalis*]	ACA50924.1	0.895102
CL6671.Contig2	2.499921	631	carboxylesterase [*L. sticticalis*]	ACA50924.1	0.811204
Unigene15398	3.088288	212	putative trypsin 11 [*Ostrinia nubilalis*]	AFM77759.1	0.831683
CL5516.Contig2	2.583776	775	trypsin serine protease [*O. nubilalis*]	ABF47507.1	0.827059
CL2723.Contig1	3.699587	327	putative trypsin 11 [*O. nubilalis*]	AFM77759.1	0.85988
CL2995.Contig2	3.334626	214	putative chymotrypsin 10 [*O. nubilalis*]	AFM77769.1	0.858833
CL2995.Contig3	3.797801	982	putative chymotrypsin 10 [*O. nubilalis*]	AFM77769.1	0.87683
CL77.Contig4	3.097627	1114	putative chymotrypsin 8 [*O. nubilalis*]	AFM77767.1	0.818677
Unigene20090	2.501993	915	putative chymotrypsin 12 [*O. nubilalis*]	AFM77771.1	0.823148
CL2995.Contig1	4.579367	1002	putative chymotrypsin 10 [*O. nubilalis*]	AFM77769.1	0.893699
CL5098.Contig1	3.322195	942	putative chymotrypsin 11 [*O. nubilalis*]	AFM77770.1	0.867685
Unigene36951	2.796642	274	chymotrypsin-like protease [*Helicoverpa armigera*]	CAA72951.1	0.837266
CL3709.Contig6	2.632878	3527	aminopeptidase N [*Ostrinia furnacalis*]	ABQ51393.1	0.802879
Unigene9047	2.781552	3082	Cry1Ab-RR resistance protein APN2 [*O. furnacalis*]	ACF34999.1	0.833355
Unigene33230	2.473809	582	aminopeptidase N3 [*O. nubilalis*]	AEO12689.1	0.804153
CL9114.Contig2	5.651439	3081	Cry1Ab resistance protein APN4 [*O. furnacalis*]	ACF34998.2	0.89408
Unigene24705	−12.0969	740	cadherin-like protein gene, complete cds [*O. nubilalis*]	DQ000165.1	0.887516
CL30.Contig5	−3.84577	844	cadherin-like protein gene, complete cds [*O. nubilalis*]	DQ000165.1	0.854111
Unigene31030	−8.42642	308	cadherin-like protein gene, complete cds [*O. nubilalis*]	DQ000165.1	0.921197
Unigene6834	−3.07792	1761	cadherin-like protein gene, complete cds [*O. nubilalis*]	DQ000165.1	0.834118
CL7354.Contig2	−2.68449	2059	alkaline phosphatase D	K01113	0.826474
Unigene7296	−8.4793	1122	beta-actin [*Dugesia japonica*]	AFX73037.1	0.938707
CL4610.Contig1	−4.54035	1259	V-ATPase subunit A [*O. furnacalis*]	ADP23923.1	0.88797
Unigene14903	−8.28497	2037	heat shock protein 70 [*Homarus americanus*]	ABA02165.1	0.935031
Unigene5163	−8.03265	917	heat shock protein 70 [*Spodoptera exigua*]	ACQ78180.1	0.870403
Unigene25984	−8.32476	1076	ABC transporter family protein [*Tetrahymena thermophila*]	XP_977039.1	0.916564
Unigene13233	−12.1344	493	ABC transporter B family protein [*Polysphondylium pallidum*]	EFA77764.1	0.890445

*Limitations of all significantly different expressed genes between Cry1Ab susceptible strain (ACB-BtS) and resistant strain (ACB-AbR) of *Ostrinia furnacalis* are based on *Q*-value < 1 and the absolute value of log_2_Ratio ≥ 1. The log_2_Ratio indicates the change of gene expression; a positive number means up-regulation and a negative one means down-regulation.

**Figure 5:**
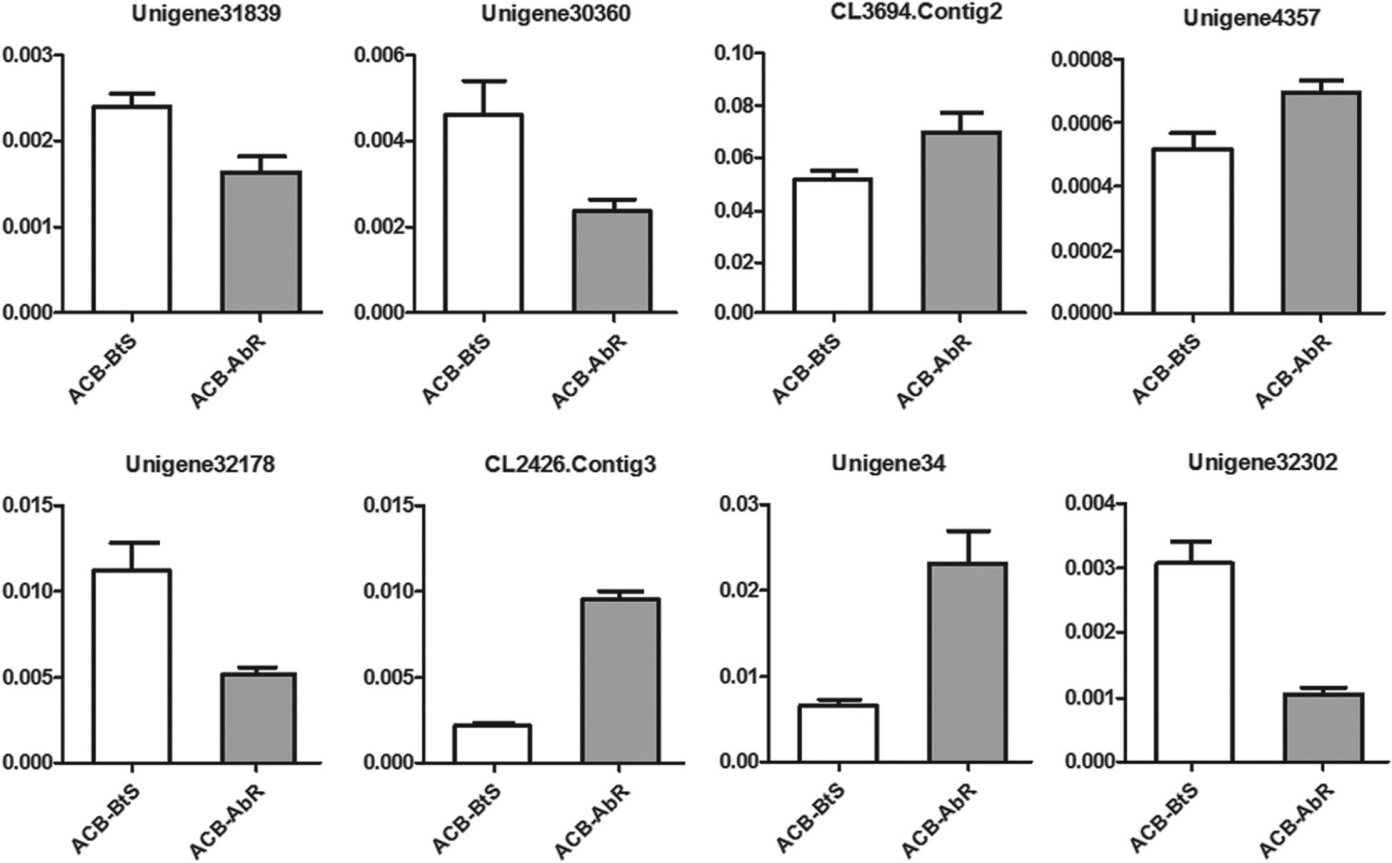
**RT-qPCR analysis of eight randomly selected genes undertaken to confirm expression patterns indicated by the sequencing.** Quantitative real-time PCR analysis data from 8 select genes are presented. Three technical replicates were performed for each of three biological replicates. The height of each box represents the mean average of sample-specific 2^-ΔΔCt^ values.

To verify the gene expression patterns that were observed in the sequencing data, RT-qPCR analyses were performed on eight randomly selected genes; Unigenes 30360, 31839, 32178, 32302 and 4357, and CL3694.Contig2, CL2426.Contig3, and β-actin as the candidate reference gene for RT-qPCR normalization. These analyses (Figure 5) supported the DEU data. The expression of Unigenes 30360, 31839 and 32178, 32302 was higher in ACB-BtS, whereas the expression of CL3694.Contig2, CL2426.Contig3, Unigenes34570 and 4357 was higher in ACB-AbR. This high confirmation rate indicated the reliability of the data.

### Function analysis of differentially expressed unigenes

**Figure 6:**
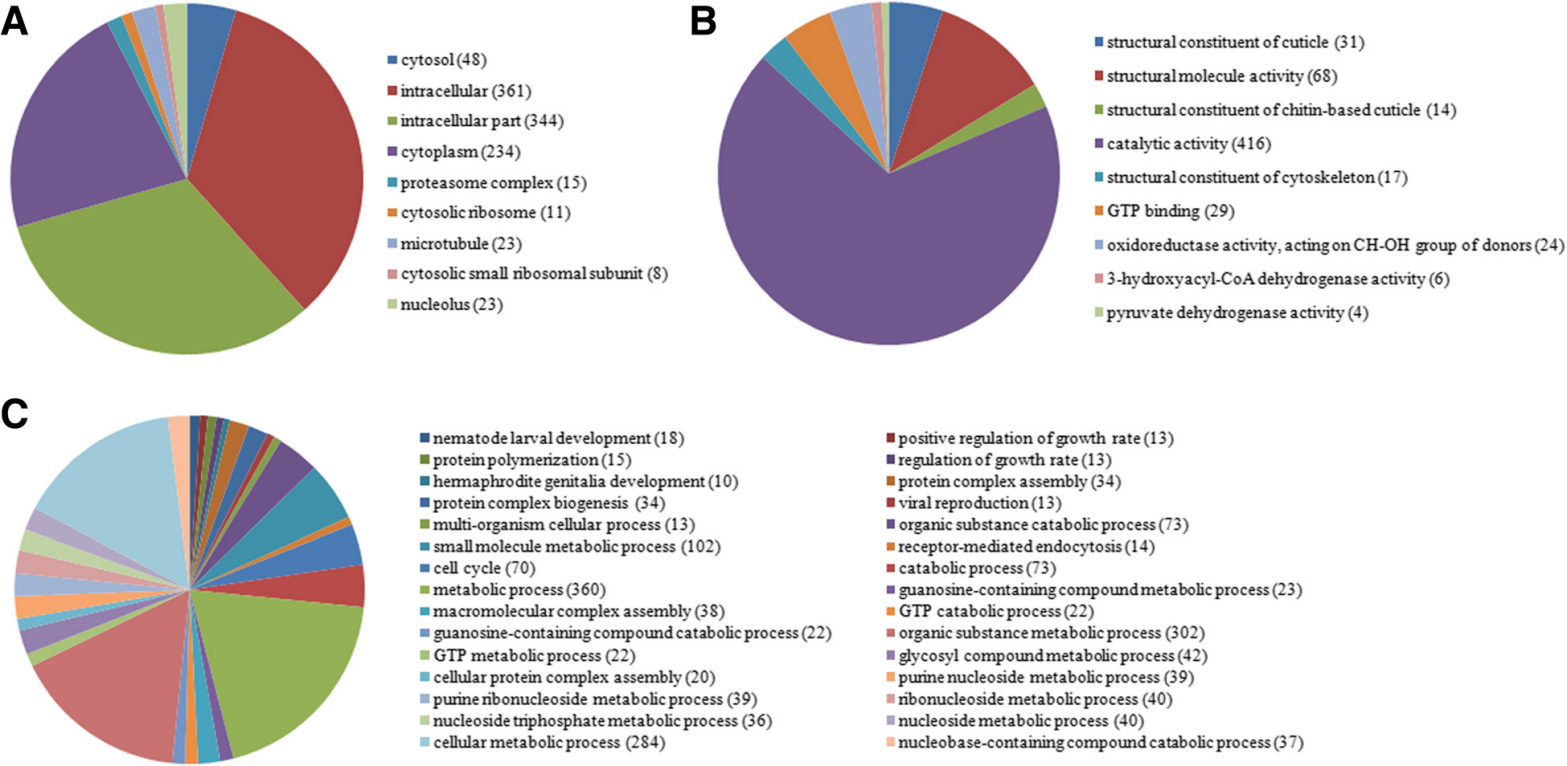
**Categories of significantly enriched GO terms for the differentially expressed unigenes (DEUs) between Cry1Ab susceptible (ACB-BtS) and Cry1Ab resistant (ACB-AbR)** ***Ostrinia furnacalis*** **. A.** Cellular components. **B.** Molecular functions, and **C.** Biological processes. (Numbers of DEUs).

To explore the biological function of the significant DEUs between ACB-BtS and ACB-AbR, GO functional and pathway enrichment were analyzed. Four hundred and twenty, 614 and 562 DEUs were annotated to 222, 319 and 1369 GO terms of cellular component, molecular function, and biological process, respectively (corrected *P*-value ≤1, Additional file 9: Table S6-8). The DEUs were significantly enriched to nine cellular component categories (corrected *P*-value ≤0.05), in which cytosol (G0: 0005829, corrected *P*-value = 1.45e^−08^) was most strong presented and the category, intracellular (GO: 0005622), was the largest with 361 DEUs (Figure 6).

The DEUs were significantly enriched to nine molecular function categories of mainly two types: structural constituent of cuticle and enzymatic activity. Under the significantly enriched GO terms, 48 DEUs were annotated to the GO terms associated with cuticle/cytoskeleton. Of these, 47 DEUs were down-regulated in ACB-AbR. The GO term associated with catalytic activity, which accounted for 67.8% of the transcripts involved in these GO terms, was the largest proportion of the molecular function terms. A total of 416 unigenes were annotated under this term, and 85.8% were down-regulated in ACB-AbR. In addition, six unigenes annotated to aminopeptidase activity and two to APN (CL3709.Contig6 and Unigene 9047) were up-regulated in ABC-AbR. All others were down-regulated. However, the DEUs, with up-regulated expression in ACB-AbR, represented 66.7% of the all DEUs annotated to serine type peptidase activity.

Under the significantly enriched GO terms in “Biological process”, those associated with regulation of development/growth process accounted for 5.5% of the DEUs involved in this ontology. The DEUs involved in this process were under-transcribed in ACB-AbR. In addition, GO terms associated with regulation of proteolysis, apoptotic process, cell death and regulation of immune system process were under-transcribed in ACB-AbR. In contrast, the GO term associated with regulation of vasculature development and regulation of blood coagulation were over-transcribed in ACB-AbR. The greatest proportion of the biological process terms was associated with metabolic/catabolic process, in which the largest GO term, metabolic process, covered 360 DEUs, where 321 DEUs were down-regulated in ACB-AbR, while 39 DEUs were up-regulated.

Metabolic pathway enrichment analysis demonstrated that 1423 DEUs were involved in 236 pathways (Additional file 10: Table S9) which potentially contribute to ACB Cry1Ab resistance. Of these, 37 functional pathways were significantly enriched (*Q*-value < 0.05), five of which have been associated with infection, including *vibrio cholerae* infection (ko05110), pathogenic *Escherichia coli* infection (ko05130), *staphylococcus aureus* infection (ko05150), Epstein-Barr virus infection (ko05169), and Herpes simplex infection (ko05168). All DEUs, except CL6099.Contig1, enriched to the pathways associated with bacterial infection were all down-regulated in ACB-AbR. Also, there were 35 DEUs enriched to proteasome (ko03050), 34 of which were down-regulated in ACB-AbR. In addition, nine DEGs were enriched to ABC transporters (ko02010), four of which were up-regulated and five down-regulated in ACB-AbR. It was noteworthy that 18 DEGs were highly significantly enriched to GPI-anchor biosynthesis, and all genes mapping to the pathway were substantially down-regulated in ACB-AbR.

## Discussion

Although ACB is one of the main target pests of Bt transgenic maize, the mechanisms of its development of resistance to Cry1Ab and Cry1Ac [6,28] remains unclear. In this study, a laboratory strain (ACB-BtS) susceptible to all insecticides and a Cry1Ab-resistant strain (ACB-AbR) selected using Cry1Ab toxin for more than 135 generations with more than 100-fold resistance to Cry1Ab after 35 generations were investigated through RNA-Seq technology to analyze the defense response of larvae to Cry1Ab.

Due to the development of deep sequencing and improvement in characterization of many transcriptomes, RNA-Seq technology has become increasingly use for identification of the resistance-related genes in insects [29-32]. Two biological replicates of ACB-BtS and ACB-AbR, were sequenced by Illumina HiSeq^TM^ 2000 in this study. An average of 52.7 million 90 bp clean reads were generated, providing more original information than obtained in related studies, e.g. studies on *P. xylostella* [31] and *Panany chuscitri* [33], both of them generating 26 million 90 bp clean reads. The clean reads were assembled *de novo* using the short reads assembling program-Trinity [34]. For ACB, as a non-model insect without a reference genome sequence, the assembly by Trinity was better than that of other programs [35]. By efficiently constructing and analyzing sets of de Bruijn graphs, Trinity could fully reconstructs a large fraction of transcripts, including alternatively spliced isoforms and transcripts from recently duplicated genes. Through analysis of larvae, chrysalis and adult, an average of 57,245 unigenes with a mean length of 607 nt was generated for the four samples of ACB. Among them, 35,723 unigenes could be matched using NR, NT, Swiss-prot, KEGG, COG and GO databases. Specifically, the NR database had 30,264 (84.7%) BLAST results, was the highest number of annotated transcripts from these databases. More than 70% NR-annotated unigenes were matched with the sequences from *D. plexippus*, *B. mori,* and *T. castanuem*. This was a result of each having completely sequenced genomes (BioProject Number: PRJNA72423, PRJNA205630, PRJDA20217, PRJNA13125, PRJNA12259, PRJDA49727, PRJNA15718, PRJNA12540). Among these annotations, there were 168, 590 and 121 transcripts were homologous to candidate Bt receptors genes, cadherin-like protein, APN and ALP, respectively. The unigenes, which were annotated to glycolipid, actin, V-type proton ATPase, heat shock protein, and ABC transporter also were detected. Also, unigenes were found that matched the chymotrypsinsor trypsin-like protein in the annotation program. These data provided the foundation for gene function analysis.

The mutation of Bt receptor genes has been documented to be associated with the insect resistance to Bt [36]. A Cry1Ac-selected strain of ACB evolved three mutant alleles of a cadherin-like protein, which mapped within the toxin-binding region. Each of the three mutant alleles possessed two or three amino acid substitutions in this region [37]. Compared with APN sequences from the Bt susceptible ACB strain, there were 9, 5, 10 and 12 amino acid variations in the deduced protein sequences from the Cry1Ab resistant strain [23]. The SNPs existed on a certain site of four samples were detected through SOAPsnp software in the present study. When focusing on differences in polymorphism between ACB-BtS and ACB-AbR, 18 unigenes annotated to putative Bt receptor genes, presented SNP on the same position but different type, were detected. Among them, 2 cadherin-like proteins, 10 APNs and 1 ALP displayed differential SNPs leading to non-synonymous changes between the resistant and susceptible strains. Although this requires functional validation, this further suggests that the APN, cadherin-like protein, and ALP could be Bt receptor genes in ACB*.*

To detect the Cry1Ab resistance genes, the genes differentially expressed between ACB-BtS and ACB-AbR were analyzed using *NOISeq* [38]. Cry1Ab exposure resulted in large alterations of the ACB transcriptome profile, including 636 unigenes being up-regulated and 3,157 being down-regulated in ACB-AbR. DEG analysis indicated that a total of 1215 genes, 189 up-regulated and 1026 down-regulated, were differentially expressed between the susceptible and chlorantraniliprole-resistant *P. xyllostella* [30]. However, the study on midgut transcriptome response to Cry1Ac in *P. xylostella* indicated that the Cry1Ac resistant strains have more up-regulated than down-regulated unigenes [31]. Among the DEUs between ACB-BtS and ACB-AbR, many were associated with growth regulation and chitin. It was speculated that the different trends among experiments were caused by differences in the materials analyzed. The whole body of target insects was used in the *P. xyllostella*-chlorantraniliprole and *O. furnacalis*-Cry1Ab resistance study [30]. Whereas, midgut tissue was used in the *P. xylostella*- Cry1Ac resistance study [31].

To further verify the gene expression data, eight genes, four with increased and four with decreased expression in ACB-AbR, were selected for RT-qPCR. The RT-qPCR results agreed with the DEU data, providing confidence in reliability of our data.

Two mechanisms of resistance to Bt toxins have been described in insects: (1) altered protoxin activation by gut proteases, and (2) modification in transcription level and/or protein sequence of Cry receptors resulting in lower of failure in toxin binding [39-41]. Compared to ACB-BtS, annotated proteases, chymotrypsin/trypsin, were over-transcribed in ACB-AbR. The over-transcription of several proteases and under-transcription of detoxification enzymes were in accordance with observation in a Bti resistant mosquito strain [32]. The increased proteolytic activity in ACB-AbR could reflect a higher ability to degrade toxins.

Different isoforms of APNs and cadherin-like protein together with ALP have been shown to interact with different types of Cry toxins [36,42,43]. In this study, dozens of unigenes, annotated to APN, and cadherin-like protein were differentially expressed in ACB-AbR. It has been hypothesized that the Cry1Ab resistance in the ACB is correlated with the up-regulation of APN1, APN2, APN3 and APN4 [23]. Similarly, in this study, the CL3709.Contig 6, Unigene 9047, Unigene33230 and CL9114.Contig2 annotated to complete cds of APN1 (ABQ51393.1), APN2 (ACF34999.1), APN3 (AEO12689.1) and APN4 (ACF34998.2), respectively, were significantly up-regulated (2.47 to 5.65-times) in ACB-AbR. Whereas, there was no significant difference was detected in the expression of the unigenes annotated to *O. nubilalis* (APN5, AEO12694.1; APN7, AEO12692.1 and; APN8, AEO12693.1), *T. ni* (APN6, AEA29694.1), and *B. mori* (APN9, AFK85025.1; APN10, AFK85026.1; APN11, AFK85027.1 and APN14, AFK85030.1). Biochemical, proteomic, and molecular analyses showed that the Cry1Ac resistance of *T. ni* was correlated with down-regulation of APN1. Also, the concurrent up-regulation of APN6 might, in part, compensate for the loss of APN1 to minimize the fitness cost of resistance [44]. It was also reported that the APN encoded by the Unigene26057-mk was significantly down-regulated (2.10-times), whereas Unigene59183-mk was significantly up-regulated (2.31-times) in the resistant MK *P. xyostella* [31]. The total APN proteolytic activity and gene expression of APN1, APN2 and APN3 from Cry1Ab resistance *Diatraea saccharalis* were significantly lower than those of the Cry1Ab susceptible strain [45].

Meanwhile, four unigenes (Unigene24705, CL30.Contig5, Unigene31030 and Unigene6834l) annotated to cadherin-like protein (DQ000165.1) were down-regulated in ACB-AbR. These results agreed with a previous study on cadherin-like expression difference in Cry1Ac resistance ACB [37]. The transcript abundance of a midgut cadherin-like protein (DsCAD1) of *D. saccharalis* was significantly lower in Cry1Ab resistant strain, and the down-regulation of DsCAD1 expression by RNAi was functionally correlated with a decrease in Cry1Ab susceptibility [46]. However, expression of Unigene32060-mk and Unigenen38756-mk, which were annotated to be cadherin-like proteins, were highly elevated in the resistant MK *P. xyostella* [31]. It was noteworthy that not all unigenes with the same annotation had the same expression pattern. For example, the unigene (CL8115.Contig1) also was annotated to cadherin-like protein (DQ000165.1) was up-regulated in ACB-AbR. It was speculated that this was a unigene associated with cadherin-like protein, but not the gene itself.

Given the molecular characterization and the capability of GPI-anchored ALP to bind to the Bt toxin [47], the ALPs isolated from lepidopteran and dipteran species have been identified as receptors for Cry1Ac [15,42], Cry11Aa [47] and Cry4Ba toxins [48]. In total, 121 transcripts were annotated to ALP in this study, however, no DEUs annotated to ALP were detected in ACB-AbR. Only one unigene (CL7354.Contig2) associated to ALP pathway was under-transcripted (−2.68) in ACB-AbR.

Based on the pore-formation model, the expression of Bt receptor genes should be down-regulated in resistant insects [49,50], however, the current results were not always consistent with this. Combined with the previous research, we speculated that the APN and cadherin-like protein should have a central role in Cry1Ab resistance of ACB. However, further functional studies are needed to reveal the exact mode of action of its Bt receptor genes.

To find other Cry1Ab resistance related genes in ACB, GO function and KEGG pathway enrichment were analyzed for the DEUs of ACB-BtS and ACB-AbR. The evolution of insect resistance to Bt toxins involves selection of recessive or dominant resistance genes and their interactions, including fitness costs [51]. The overall fitness cost was closely linked to egg hatching rate, fecundity, emergence rate, larval survival rate and developmental duration of adults [52]. The developmental time of ACB-AbR larvae has been reported to be longer than ACB-BtS and the survival of ACB-AbR reduced [53]. Moreover, the number of eggs deposited by ACB-AbR was significantly lower than ACB-BtS [53]. Similarly, the analysis of specific GO categories for DEUs between ACB-BtS and ACB-AbR in the current research showed a significant decreased expression of unigenes related to cuticle/cytoskeleton and development/growth process in ACB-AbR, suggesting a fitness tradeoff between growth and resistance development.

The analysis of the GO categories of the DEUs showed that a significant portion was involved predominantly in metabolic and catabolic processes. Specifically, the catalytic activity category in the molecular function domain was represented by 416 DEUs between ACB-BtS and ACB-AbR. Similar dominance of catalytic genes was also observed in the midgut transcriptome of *D. saccharalis* [54], *H. virescens* [55] and *P. xylostlla* [31]. However, the majority of these DEUs (85.8%) were down-regulated in ACB-AbR, unlike the discovery in *P. xylostlla*, in which majority of the DEUs were up-regulated in Cry1Ac resistant stain [31]. These findings suggested that the mechanism of Cry1Ab resistance in ACB might differ from that in *P. xylostlla,* or the up-regulated expression of the minority unigenescould be compensating for the lose of the other catalytic genes to minimize the fitness costs of the resistance. As reported, P450, CaE, GST, superoxide dismutase (SOD), and prophenoloxidase (PPO) were related to the isecticide’s metabolism. Compared to the Bt susceptible ACB strain, the activity of α-naphthylacetate esterase (CarE) was higher in the Cry1Ab resistant strain, however, no significant difference was detected in acetylcholine esterase (AchE) between the Cry1Ab susceptible and resistant strains [56]. It was reported that Cry1Ac could enhance the activity of AchE in ACB, while weakening the activity of CarE, CaE, and GST [57]. In the present study, the expression level of unigenes annotated to GST and P450 was lower in ACB-AbR, whereas, the two unigenes annotated to CaE were up-regulated. However, no difference was observed in the expression of CarE and AchE.

Pathway analysis indicated that 1423 DEGs were involved in 236 pathways including energy, reproduction, microorganism infection, drug metabolism and disease pathways. The ABC transporter pathway, previously found to be related to Bt resistance [20,21], was interconnected with the entire enriched network [31]. ACB transporter comprises seven subfamilies, three of them, ABCB, ABCC and ABCG, were involved in drug resistance [58]. Previous studies have liked ABCC2 with Cry1Ac resistance in three lepidopterans [20,21]. In *P. xylostella*, a mutated ABCC2 resulted in the failure of Cry1Ac to bind to membrane vesicles, which leads to Bt resistance. In the transcriptome of *P. xylostella*, eight unigenes from ABCC2 were detected in the Cry1Ac resistant strain, and majority of them were down-regulated [31]. In this study, differentially ACB transporters between ACB-BtS and ACB-AbR included ABCB1, ABCB7, ABCB8 and ABCG1. Among them, four unigenes (CL9071.Contig1, CL8310.Contig1, CL1226.Contig5, Unigene18584) associated with ABCB1 were up regulated in ACB-AbR (3.2 to 11.9 times), the others were down-regulated.

Lipid raft, which are member domains enriched in GPI-anchored proteins, are suggested to be central in Bt-toxins toxicity. The specific DEUs associated with this mechanism was not detected in this study. However, it was worth noting that 18 DEGs were involved in GPI-anchor biosynthesis, and all genes mapped to the pathway were substantially down-regulated (6.6 to 13.0 times) in ACB-AbR. APNs have shown to attach to the membrane by a GPI anchor, which caused the toxin-binding APN to form a tight aggregate with other proteins in brush border membrane preparation solubilized by non-ionic detergents [36,59]. We speculated that the down-regulation of GPI-anchor biosynthesis in ACB-AbR reduced the binding between APN with membrane, which loosen the aggregation of Bt toxin in the BBMV, even some APNs were up-regulated in ACB-AbR. However, further functional studies are required to prove whether the change of transcriptome in ACB-AbR contributes to the Cry1Ab resistance of ACB.

In addition, we noticed there were 489 DEUs (34.4%) implicated in disease pathways and microorganism infection, including amoebiasis, Parkinson’s disease, Huntington’s disease, maturity onset diabetes of the young and *Vibrio cholera* infection, Pathogenic *Escherichia coli* infection, *Staphylococcus aureus* infection and Epstein-Barr virus infection. Of these DEUs, 95.9% were down-regulated, but only 4.1% were up-regulated in the ACB-AbR.

## Conclusions

In conclusion, this study is the first to report genetic information on ACB from sequenced transcriptome and constructed DEG libraries. This study revealed a large number of genes, which have greatly enriched sequence information for ACB. We identified genes that are potential candidates for conferring Bt resistance in ACB. This not only included the classical candidate Bt genes, such as APN and a cadherin-like protein, but also detected the novel genes encoding proteins involved in growth, metabolic and GPI-anchor biosynthesis. Through this research, we postulate that resistance to Bt toxins of ACB is a complex process involving an array of genetic and metabolic factors. With these important genetic resources, we plan to further validate the gene functions associated with Bt resistance in ACB using RNA interference (RNAi) technology.

## Methods

### Asian corn borer rearing and resistant strain selection

The laboratory strain of ACB was originally collected from a summer corn field of central China. It was maintained at 27 ± 1°C, 70-80% relative humidity (RH) and a 16:8 (L:D) photoperiod at the Institute of Plant Protection, Chinese Academy of Agricultural Sciences, Beijing. During this period the strain had no contact with any insecticides. This strain was considered to be a susceptible strain (designated ACB-BtS). Basing on the ACB-BtS, trypsin-activated Cry1Ab toxin (94% pure protein) was used as a source of Cry1Ab for the selection diet. The selected strain (ACB-AbR) was initially exposed throughout larval development to Cry1Ab in the artificial diet (2.5 ng toxin /g). The toxin concentration was steadily increased in succeeding generations to target 40-70% mortality in the exposed insects. After 51 generations, ACB-AbR strain was reared on diet containing 400 ng toxin /g. ACB-AbR had developed more than 100-fold resistance to Cry1Ab after 35 generations of selection [6]. In this study, the ACB-AbR, which has been selected more than 135 generations, was used to detect the Cry1Ab resistance-relative genes in ACB. In parallel, the ACB-BtS, which was reared in the absence of any toxin, was used as the negative control strain. One individual larva from 1–5 instar larvae was collected in a PE tube as one biological replicate for both ACB-BtS and ACB-AbR. Five biological replicates for each sample were collected and processed independently. Two replicates were used in gene expression profile analysis and Illumina sequencing, and the others were used for the RT-qPCR analysis. All samples were stored at −80°C until assayed.

### RNA-seq library preparation and Illumina sequencing

The following protocols were performed by staff at the Beijing Genome Institute (BGI, Shenzhen, China). Total RNA was extracted using TRIzol reagent (Invitrogen, Carlsbad, CA, US) and treated with RNase-free DNase I. Poly(A) mRNA was isolated using oligodT beads and fragmented into small pieces in Thermomixer under elevated temperature. Double-stranded cDNA was then synthesized using the SuperScript double-stranded cDNA Synthesis kit (Invitrogen) with random hexamer (N6) primers (Illumina). These cDNA fragments then underwent an end repair process followed by phosphorylation and ligation of adapters. Products were subsequently purified and amplified by PCR to create the final cDNA libraries. Finally, after validating on an Agilent 2100 Bioanalyzer and ABI Step One Plus Real-Time PCR System, the cDNA library was sequenced on a flow cell using Illumina HiSeq2000 (San Diego, CA, USA).

### Bioinformatics analysis of the transcriptome

The sequences from the Illumina sequencing were deposited in the NCBI Sequence Read Archive (SRA). The high-quality reads were obtained by removing adaptor sequences, empty reads low-quality sequences (reads with unknown “N” > 5% sequences), and reads with more than 20% Q ≤10 base from the raw reads. Transcriptome *de novo* assembly was carried out through the short reads assembling program-Trinity [34]. The high-quality reads were loaded into the computer, and a de Bruijn graph data structure was used to represent the overlap among the reads. After *de novo* assembly with Trinity, the assembled unigenes were used for BLAST searches and annotation against the NR, Swiss-prot protein database, KEGG, COG (e-value < 0.00001), and the best aligning results were used to decide direction of unigenes. If results from different databases conflicted with each other, a priority order of NR, Swiss-Prot, KEGG and COG was followed to determine the sequence direction. When the unigene was unaligned with any of the above database, ESTScan software was used to predict its coding regions and to determine its sequence direction. Next, unigene sequence were firstly aligned by blastx to protein databases like NR, Swiss-Prot, KEGG and COG (e-value < 0.00001), and aligned by blastn to nucleotide databases NT (e-value < 0.0001), retrieving proteins with the highest sequence similarity with the given unigenes along with their protein functional annotations. With NR annotation, Blast2GO program [60] was used to get GO annotation and KEGG pathway of uinigenes. In the last step, SOAPsnp software (soap.genomics.org.cn) was used to identify SNP between reads from specific strain to all unigenes.

### Differential gene expression in resistant and susceptible Asian corn borer

The FPKM method is able to eliminate the influence of different gene length and sequencing level on the calculation of gene expression. Therefore the calculated gene expression was directly used for comparing the difference of gene expression between samples.

The FPKM between the biological replications was analyzed by Pearson correlation. The Pearson coefficient of unigene expression in different replications is more than 0.85, indicating consistency between the replicates. If the value of either sample FPKM was zero, 0.01 was used to instead of 0 to calculate the fold change. According to the correlation results, the *NOISeq* software was selected to analyze differential expression between ACB-AbR and ACB-BtS with options *Q*-value ≥ 0.8, ralative change ≥ 2. Through SOAP software, reads were mapped to the reference, under the condition like this insert size = 400 to 600 bp and maximum number of mismatches allowed on a read = 5 bp.

### Real-time quantitative PCR analysis of gene expression

The transcriptome results were verified using RT-qPCR. Total RNA used for RT-qPCR analysis was extracted from the mixture of 1–5 instar larvae from ACB-BtS and ACB-AbR, using three biological replicates respectively. Total RNA was extracted as described above, genomic DNA was removed with DNase I, and total RNA concentration was measured. First-strand cDNA was synthesized from 4 ug of DNA-free RNA using the M-MLV Rtase (Thermo, USA) according to the manufacture’s instructions. The cDNA was used as the template for RT-qPCR. Primer sequences were listed in Additional file 11: Table S10. The RT-qPCR mixture (25 μl total volume) contained 12.5 μl of SYBRGreen Mix (Thermo, USA), 0.5 μl of each primer (10 μM), 2 μl cDNA, and 9.5 μl of RNase-free water. The reactions were performed on ABI 7300 Real-time RCR according to the manufacture’s instructions. The RT-qPCR program began with 10 min at 95°C, followed by 40 cycles of 95°C for 15 s and 60°C for 45 s, then ended with 95°C for 15 s; 60°C for 1 min; 95°C for 15 s; and 60°C for 15 s. cDNA-less controls for each primer pair were included in each run. Expression was calculated as 2^-ΔΔCt^ and normalized to that of the reference gene.

### Function analysis of differentially expressed unigenes

Go functional analysis provides GO functional classification annotation for DEUs as well as GO functional enrichment analysis. The annotation terms form the GO ontology were obtained from Blast2GO [60] by first mapping all DEUs to each term of Gene ontology database (www. geneontology. org) and then calculating the gene numbers for each GO term. The hypergeometric test was applied to this list of gene and numbers for each GO term to find significantly enriched GO terms in DEUs compared to the transcriptome background. The *p*-value for the hypothesis test was calculated with the formula:

$$ P=1-{\displaystyle \sum_{i=0}^{m-1}\frac{\left[\begin{array}{l}M\\ {}i\end{array}\right]\left[\begin{array}{l}N-M\\ {}n-i\end{array}\right]}{\left[\begin{array}{l}N\\ {}n\end{array}\right]}} $$

Where N is the number of all genes with GO annotation; n is the number of DEUs in N; M is the number of all genes that are annotated to the certain GO terms; m is the number of DEUs in M.

The calculated *p*-value then underwent Bonferroni Correction, taking corrected *p*-value ≤ 0.05 as a threshold. GO terms fulfilling this condition are defined as significantly enriched GO terms in DEUs. The analysis is able to recognize the main biological functions that DEUs exercise. Meanwhile, the GO functional enrichment analysis also integrates the clustering analysis of expression patterns. Thus, allowing the expression patterns of DEUs annotated to the given GO-term.

Pathway based analysis helps to further understand genes biological functions. KEGG is the major public pathway related database [40]. Pathway enrichment analysis identifies significantly enriched metabolic pathways or signal transduction pathways in DEUs comparing with the whole transcriptome background. The formula for the *p*-value is similar to that of the GO analysis. Here N is the number of all genes that with KEGG annotation, n is the number of DEUs in N, M is the number of all genes annotated to specific pathways, and m is number of DEUs in M.

### Availability of supporting data

SRP046207: Sequence Read Archive, National Center for Biothechnology Information, http://www.ncbi.nlm.nih.gov/sra/?term=SRP046207.

## Additional files

Additional file 1: Figure S1. Length distribution of contigs in the two replicates from Cry1Ab susceptible (ACB-BtS) and resistant (ACB-AbR) strains of *Ostrinia furnacalis.*
Additional file 2: Figure S2. Length distribution of unigens in the two replicates from Cry1Ab susceptible (ACB-BtS) and resistant (ACB-AbR) strains of *Ostrinia furnacalis.*
Additional file 3: Table S1. Annotation of all uniques from *Ostrinia furnacalis* samples. Additional file 4: Figure S3. Characteristics of homology search of *Ostrinia furnacalis* unigenes against the NR database. (A) E-value distribution of the top BLAST hits for each unique sequence. (B) Similarity distribution of the top BLAST hits for each unique sequence. (C) Species distribution of the top BLAST hits for all homologous sequences. Additional file 5: Table S2 Unigenes with a COG classification. Additional file 6: Table S3. All unigenes which were categorized into GO terms. Additional file 7: Table S4. The unigenes which were mapped into KEGG pathways. Additional file 8: Table S5. The SNPs existed in the same site of *Ostrinia furnacalis* samples. Additional file 9: Table S6-S8. GO annotation of DEUs between Cry1Ab susceptible and resistant *Ostrinia furnacalis.*
Additional file 10: Table S9. Metabolic pathway enrichment analysis of DEUs between Cry1Ab susceptible and resistant *Ostrinia furnacalis*. Additional file 11: Table S10. Primers used for q-PCR.
