# Supplementary material for: Transcriptome differences between Cry1Ab resistant and susceptible strains of Asian corn borer
Source: BMC Genomics. 2015 Mar 12;16(1):173. doi: 10.1186/s12864-015-1362-2 (PMC4406038; doi:10.1186/s12864-015-1362-2)
Supplement: Additional file 1: Figure S1. — Length distribution of contigs in the two replicates from Cry1Ab susceptible (ACB-BtS) and resistant (ACB-AbR) strains of Ostrinia furnacalis. [file 12864_2015_1362_MOESM1_ESM.docx]

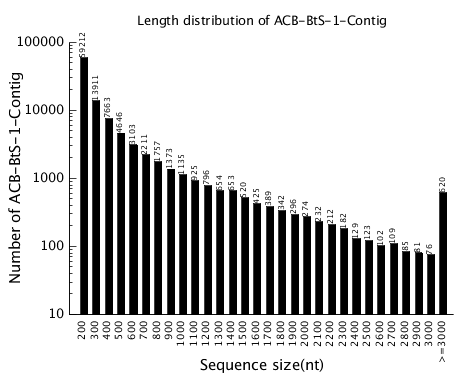


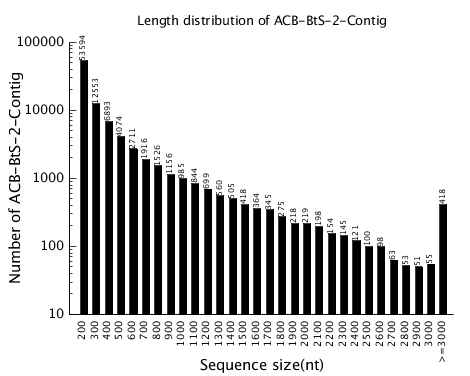


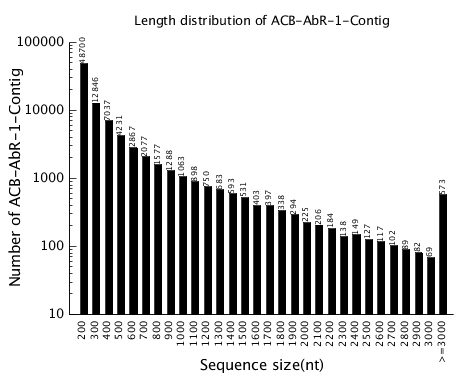


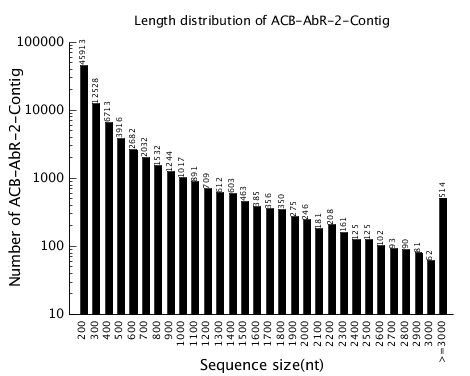


Figure S1 Length distribution of contigs in the two replicates from Cry1Ab susceptible (ACB-BtS) and resistant (ACB-AbR) strains of *Ostrinia furnacalis*
