# Supplementary figures and images for: Transcriptome differences between Cry1Ab resistant and susceptible strains of Asian corn borer
Source: BMC Genomics. 2015 Mar 12;16(1):173. doi: 10.1186/s12864-015-1362-2 (PMC4406038; doi:10.1186/s12864-015-1362-2)

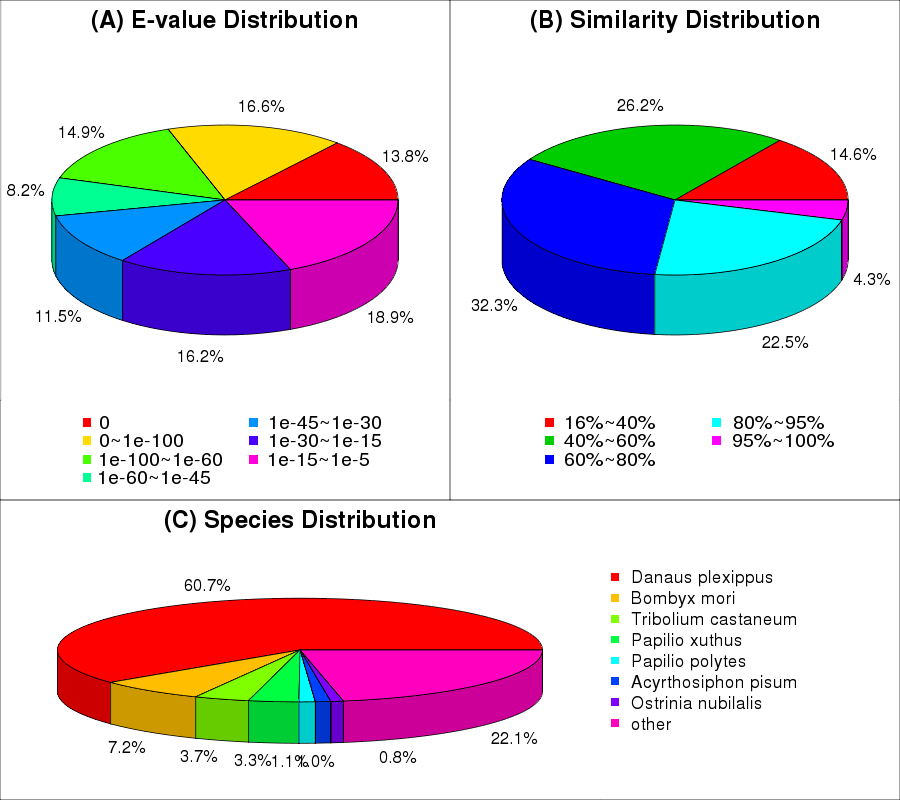

Supplement: Additional file 4: Figure S3. — Characteristics of homology search of Ostrinia furnacalis unigenes against the NR database. (A) E-value distribution of the top BLAST hits for each unique sequence. (B) Similarity distribution of the top BLAST hits for each unique sequence. (C) Species distribution of the top BLAST hits for all homologous sequences. [file 12864_2015_1362_MOESM4_ESM.tiff]
