# Supplementary material for: Transcriptome differences between Cry1Ab resistant and susceptible strains of Asian corn borer
Source: BMC Genomics. 2015 Mar 12;16(1):173. doi: 10.1186/s12864-015-1362-2 (PMC4406038; doi:10.1186/s12864-015-1362-2)
Supplement: Additional file 10: Table S9. — Metabolic pathway enrichment analysis of DEUs between Cry1Ab susceptible and resistant Ostrinia furnacalis. [file 12864_2015_1362_MOESM10_ESM.docx]

Table S9 Metabolic pathway enrichment analysis of DEUs between Cry1Ab susceptible and resistant *Ostrinia furnacalis*

| Number | Pathway | DEGs with pathway annotation (1423) | All genes with pathway annotation (20144) | Pvalue | Qvalue | Pathway ID |
| --- | --- | --- | --- | --- | --- | --- |
| 1 | [Ribosome](file:///D:\%E5%8D%8E%E5%A4%A7%E8%BD%AC%E5%BD%95%E7%BB%84%E6%95%B0%E6%8D%AE\GeneDiffExp\GeneDiffExp\Pathway\ACB-BtS-VS-ACB-AbR.html#gene1) | 74 (5.2%) | 188 (0.93%) | 1.41593e-36 | 3.341595e-34 | ko03010 |
| 2 | [Vibrio cholerae infection](file:///D:\%E5%8D%8E%E5%A4%A7%E8%BD%AC%E5%BD%95%E7%BB%84%E6%95%B0%E6%8D%AE\GeneDiffExp\GeneDiffExp\Pathway\ACB-BtS-VS-ACB-AbR.html#gene2) | 124 (8.71%) | 518 (2.57%) | 9.741459e-35 | 1.149492e-32 | ko05110 |
| 3 | [Amoebiasis](file:///D:\%E5%8D%8E%E5%A4%A7%E8%BD%AC%E5%BD%95%E7%BB%84%E6%95%B0%E6%8D%AE\GeneDiffExp\GeneDiffExp\Pathway\ACB-BtS-VS-ACB-AbR.html#gene3) | 116 (8.15%) | 590 (2.93%) | 1.768622e-24 | 1.391316e-22 | ko05146 |
| 4 | [Proteasome](file:///D:\%E5%8D%8E%E5%A4%A7%E8%BD%AC%E5%BD%95%E7%BB%84%E6%95%B0%E6%8D%AE\GeneDiffExp\GeneDiffExp\Pathway\ACB-BtS-VS-ACB-AbR.html#gene4) | 35 (2.46%) | 88 (0.44%) | 3.728699e-18 | 2.199932e-16 | ko03050 |
| 5 | [Pathogenic Escherichia coli infection](file:///D:\%E5%8D%8E%E5%A4%A7%E8%BD%AC%E5%BD%95%E7%BB%84%E6%95%B0%E6%8D%AE\GeneDiffExp\GeneDiffExp\Pathway\ACB-BtS-VS-ACB-AbR.html#gene5) | 50 (3.51%) | 300 (1.49%) | 1.131708e-08 | 5.341662e-07 | ko05130 |
| 6 | [Glycosylphosphatidylinositol(GPI)-anchor biosynthesis](file:///D:\%E5%8D%8E%E5%A4%A7%E8%BD%AC%E5%BD%95%E7%BB%84%E6%95%B0%E6%8D%AE\GeneDiffExp\GeneDiffExp\Pathway\ACB-BtS-VS-ACB-AbR.html#gene6) | 18 (1.26%) | 64 (0.32%) | 2.719338e-07 | 1.069606e-05 | ko00563 |
| 7 | [Staphylococcus aureus infection](file:///D:\%E5%8D%8E%E5%A4%A7%E8%BD%AC%E5%BD%95%E7%BB%84%E6%95%B0%E6%8D%AE\GeneDiffExp\GeneDiffExp\Pathway\ACB-BtS-VS-ACB-AbR.html#gene7) | 18 (1.26%) | 66 (0.33%) | 4.515163e-07 | 1.522255e-05 | ko05150 |
| 8 | [Aminoacyl-tRNA biosynthesis](file:///D:\%E5%8D%8E%E5%A4%A7%E8%BD%AC%E5%BD%95%E7%BB%84%E6%95%B0%E6%8D%AE\GeneDiffExp\GeneDiffExp\Pathway\ACB-BtS-VS-ACB-AbR.html#gene8) | 27 (1.9%) | 135 (0.67%) | 7.208415e-07 | 2.126482e-05 | ko00970 |
| 9 | [Oxidative phosphorylation](file:///D:\%E5%8D%8E%E5%A4%A7%E8%BD%AC%E5%BD%95%E7%BB%84%E6%95%B0%E6%8D%AE\GeneDiffExp\GeneDiffExp\Pathway\ACB-BtS-VS-ACB-AbR.html#gene9) | 40 (2.81%) | 250 (1.24%) | 9.58859e-07 | 2.514341e-05 | ko00190 |
| 10 | [Phagosome](file:///D:\%E5%8D%8E%E5%A4%A7%E8%BD%AC%E5%BD%95%E7%BB%84%E6%95%B0%E6%8D%AE\GeneDiffExp\GeneDiffExp\Pathway\ACB-BtS-VS-ACB-AbR.html#gene10) | 52 (3.65%) | 367 (1.82%) | 1.197660e-06 | 2.826478e-05 | ko04145 |
| 11 | [Gap junction](file:///D:\%E5%8D%8E%E5%A4%A7%E8%BD%AC%E5%BD%95%E7%BB%84%E6%95%B0%E6%8D%AE\GeneDiffExp\GeneDiffExp\Pathway\ACB-BtS-VS-ACB-AbR.html#gene11) | 27 (1.9%) | 148 (0.73%) | 4.592553e-06 | 9.853114e-05 | ko04540 |
| 12 | [Epstein-Barr virus infection](file:///D:\%E5%8D%8E%E5%A4%A7%E8%BD%AC%E5%BD%95%E7%BB%84%E6%95%B0%E6%8D%AE\GeneDiffExp\GeneDiffExp\Pathway\ACB-BtS-VS-ACB-AbR.html#gene12) | 77 (5.41%) | 662 (3.29%) | 1.023886e-05 | 2.013642e-04 | ko05169 |
| 13 | [Parkinson's disease](file:///D:\%E5%8D%8E%E5%A4%A7%E8%BD%AC%E5%BD%95%E7%BB%84%E6%95%B0%E6%8D%AE\GeneDiffExp\GeneDiffExp\Pathway\ACB-BtS-VS-ACB-AbR.html#gene13) | 34 (2.39%) | 224 (1.11%) | 1.929272e-05 | 3.502371e-04 | ko05012 |
| 14 | [Amino sugar and nucleotide sugar metabolism](file:///D:\%E5%8D%8E%E5%A4%A7%E8%BD%AC%E5%BD%95%E7%BB%84%E6%95%B0%E6%8D%AE\GeneDiffExp\GeneDiffExp\Pathway\ACB-BtS-VS-ACB-AbR.html#gene14) | 27 (1.9%) | 172 (0.85%) | 7.458876e-05 | 1.257353e-03 | ko00520 |
| 15 | [Huntington's disease](file:///D:\%E5%8D%8E%E5%A4%A7%E8%BD%AC%E5%BD%95%E7%BB%84%E6%95%B0%E6%8D%AE\GeneDiffExp\GeneDiffExp\Pathway\ACB-BtS-VS-ACB-AbR.html#gene15) | 64 (4.5%) | 600 (2.98%) | 0.0006312414 | 9.853266e-03 | ko05016 |
| 16 | [Protein export](file:///D:\%E5%8D%8E%E5%A4%A7%E8%BD%AC%E5%BD%95%E7%BB%84%E6%95%B0%E6%8D%AE\GeneDiffExp\GeneDiffExp\Pathway\ACB-BtS-VS-ACB-AbR.html#gene16) | 10 (0.7%) | 43 (0.21%) | 0.000668018 | 9.853266e-03 | ko03060 |
| 17 | [Rheumatoid arthritis](file:///D:\%E5%8D%8E%E5%A4%A7%E8%BD%AC%E5%BD%95%E7%BB%84%E6%95%B0%E6%8D%AE\GeneDiffExp\GeneDiffExp\Pathway\ACB-BtS-VS-ACB-AbR.html#gene17) | 16 (1.12%) | 94 (0.47%) | 0.0008516072 | 1.182231e-02 | ko05323 |
| 18 | [Basal transcription factors](file:///D:\%E5%8D%8E%E5%A4%A7%E8%BD%AC%E5%BD%95%E7%BB%84%E6%95%B0%E6%8D%AE\GeneDiffExp\GeneDiffExp\Pathway\ACB-BtS-VS-ACB-AbR.html#gene18) | 21 (1.48%) | 141 (0.7%) | 0.0009125511 | 1.196456e-02 | ko03022 |
| 19 | [Glutathione metabolism](file:///D:\%E5%8D%8E%E5%A4%A7%E8%BD%AC%E5%BD%95%E7%BB%84%E6%95%B0%E6%8D%AE\GeneDiffExp\GeneDiffExp\Pathway\ACB-BtS-VS-ACB-AbR.html#gene19) | 22 (1.55%) | 155 (0.77%) | 0.001335110 | 1.658347e-02 | ko00480 |
| 20 | [Protein processing in endoplasmic reticulum](file:///D:\%E5%8D%8E%E5%A4%A7%E8%BD%AC%E5%BD%95%E7%BB%84%E6%95%B0%E6%8D%AE\GeneDiffExp\GeneDiffExp\Pathway\ACB-BtS-VS-ACB-AbR.html#gene20) | 50 (3.51%) | 458 (2.27%) | 0.001448171 | 1.682668e-02 | ko04141 |
| 21 | [Maturity onset diabetes of the young](file:///D:\%E5%8D%8E%E5%A4%A7%E8%BD%AC%E5%BD%95%E7%BB%84%E6%95%B0%E6%8D%AE\GeneDiffExp\GeneDiffExp\Pathway\ACB-BtS-VS-ACB-AbR.html#gene21) | 6 (0.42%) | 19 (0.09%) | 0.001497289 | 1.682668e-02 | ko04950 |
| 22 | [Ribosome biogenesis in eukaryotes](file:///D:\%E5%8D%8E%E5%A4%A7%E8%BD%AC%E5%BD%95%E7%BB%84%E6%95%B0%E6%8D%AE\GeneDiffExp\GeneDiffExp\Pathway\ACB-BtS-VS-ACB-AbR.html#gene22) | 31 (2.18%) | 270 (1.34%) | 0.005200045 | 5.578230e-02 | ko03008 |
| 23 | [Glycolysis / Gluconeogenesis](file:///D:\%E5%8D%8E%E5%A4%A7%E8%BD%AC%E5%BD%95%E7%BB%84%E6%95%B0%E6%8D%AE\GeneDiffExp\GeneDiffExp\Pathway\ACB-BtS-VS-ACB-AbR.html#gene23) | 20 (1.41%) | 154 (0.76%) | 0.006033541 | 6.190938e-02 | ko00010 |
| 24 | [Cell cycle](file:///D:\%E5%8D%8E%E5%A4%A7%E8%BD%AC%E5%BD%95%E7%BB%84%E6%95%B0%E6%8D%AE\GeneDiffExp\GeneDiffExp\Pathway\ACB-BtS-VS-ACB-AbR.html#gene24) | 43 (3.02%) | 412 (2.05%) | 0.006661435 | 6.546621e-02 | ko04110 |
| 25 | [Collecting duct acid secretion](file:///D:\%E5%8D%8E%E5%A4%A7%E8%BD%AC%E5%BD%95%E7%BB%84%E6%95%B0%E6%8D%AE\GeneDiffExp\GeneDiffExp\Pathway\ACB-BtS-VS-ACB-AbR.html#gene25) | 11 (0.77%) | 67 (0.33%) | 0.00693498 | 6.546621e-02 | ko04966 |
| 26 | [Pentose phosphate pathway](file:///D:\%E5%8D%8E%E5%A4%A7%E8%BD%AC%E5%BD%95%E7%BB%84%E6%95%B0%E6%8D%AE\GeneDiffExp\GeneDiffExp\Pathway\ACB-BtS-VS-ACB-AbR.html#gene26) | 13 (0.91%) | 96 (0.48%) | 0.01735779 | 1.575553e-01 | ko00030 |
| 27 | [Influenza A](file:///D:\%E5%8D%8E%E5%A4%A7%E8%BD%AC%E5%BD%95%E7%BB%84%E6%95%B0%E6%8D%AE\GeneDiffExp\GeneDiffExp\Pathway\ACB-BtS-VS-ACB-AbR.html#gene27) | 45 (3.16%) | 463 (2.3%) | 0.01851743 | 1.613033e-01 | ko05164 |
| 28 | [Synaptic vesicle cycle](file:///D:\%E5%8D%8E%E5%A4%A7%E8%BD%AC%E5%BD%95%E7%BB%84%E6%95%B0%E6%8D%AE\GeneDiffExp\GeneDiffExp\Pathway\ACB-BtS-VS-ACB-AbR.html#gene28) | 19 (1.34%) | 161 (0.8%) | 0.01913768 | 1.613033e-01 | ko04721 |
| 29 | [Nicotine addiction](file:///D:\%E5%8D%8E%E5%A4%A7%E8%BD%AC%E5%BD%95%E7%BB%84%E6%95%B0%E6%8D%AE\GeneDiffExp\GeneDiffExp\Pathway\ACB-BtS-VS-ACB-AbR.html#gene29) | 8 (0.56%) | 50 (0.25%) | 0.02295295 | 1.846741e-01 | ko05033 |
| 30 | [Fructose and mannose metabolism](file:///D:\%E5%8D%8E%E5%A4%A7%E8%BD%AC%E5%BD%95%E7%BB%84%E6%95%B0%E6%8D%AE\GeneDiffExp\GeneDiffExp\Pathway\ACB-BtS-VS-ACB-AbR.html#gene30) | 15 (1.05%) | 121 (0.6%) | 0.02347552 | 1.846741e-01 | ko00051 |
| 31 | [Valine, leucine and isoleucine biosynthesis](file:///D:\%E5%8D%8E%E5%A4%A7%E8%BD%AC%E5%BD%95%E7%BB%84%E6%95%B0%E6%8D%AE\GeneDiffExp\GeneDiffExp\Pathway\ACB-BtS-VS-ACB-AbR.html#gene31) | 4 (0.28%) | 17 (0.08%) | 0.02810277 | 2.139437e-01 | ko00290 |
| 32 | [Systemic lupus erythematosus](file:///D:\%E5%8D%8E%E5%A4%A7%E8%BD%AC%E5%BD%95%E7%BB%84%E6%95%B0%E6%8D%AE\GeneDiffExp\GeneDiffExp\Pathway\ACB-BtS-VS-ACB-AbR.html#gene32) | 8 (0.56%) | 55 (0.27%) | 0.03826893 | 2.822334e-01 | ko05322 |
| 33 | [Herpes simplex infection](file:///D:\%E5%8D%8E%E5%A4%A7%E8%BD%AC%E5%BD%95%E7%BB%84%E6%95%B0%E6%8D%AE\GeneDiffExp\GeneDiffExp\Pathway\ACB-BtS-VS-ACB-AbR.html#gene33) | 35 (2.46%) | 365 (1.81%) | 0.04065769 | 2.907641e-01 | ko05168 |
| 34 | [Thiamine metabolism](file:///D:\%E5%8D%8E%E5%A4%A7%E8%BD%AC%E5%BD%95%E7%BB%84%E6%95%B0%E6%8D%AE\GeneDiffExp\GeneDiffExp\Pathway\ACB-BtS-VS-ACB-AbR.html#gene34) | 2 (0.14%) | 5 (0.02%) | 0.04319804 | 2.998452e-01 | ko00730 |
| 35 | [Oocyte meiosis](file:///D:\%E5%8D%8E%E5%A4%A7%E8%BD%AC%E5%BD%95%E7%BB%84%E6%95%B0%E6%8D%AE\GeneDiffExp\GeneDiffExp\Pathway\ACB-BtS-VS-ACB-AbR.html#gene35) | 22 (1.55%) | 212 (1.05%) | 0.04512395 | 3.042643e-01 | ko04114 |
| 36 | [RNA degradation](file:///D:\%E5%8D%8E%E5%A4%A7%E8%BD%AC%E5%BD%95%E7%BB%84%E6%95%B0%E6%8D%AE\GeneDiffExp\GeneDiffExp\Pathway\ACB-BtS-VS-ACB-AbR.html#gene36) | 22 (1.55%) | 213 (1.06%) | 0.04710784 | 3.077956e-01 | ko03018 |
| 37 | [Protein digestion and absorption](file:///D:\%E5%8D%8E%E5%A4%A7%E8%BD%AC%E5%BD%95%E7%BB%84%E6%95%B0%E6%8D%AE\GeneDiffExp\GeneDiffExp\Pathway\ACB-BtS-VS-ACB-AbR.html#gene37) | 34 (2.39%) | 358 (1.78%) | 0.04825609 | 3.077956e-01 | ko04974 |
| 38 | [Lysosome](file:///D:\%E5%8D%8E%E5%A4%A7%E8%BD%AC%E5%BD%95%E7%BB%84%E6%95%B0%E6%8D%AE\GeneDiffExp\GeneDiffExp\Pathway\ACB-BtS-VS-ACB-AbR.html#gene38) | 37 (2.6%) | 398 (1.98%) | 0.05302707 | 3.208999e-01 | ko04142 |
| 39 | [Epithelial cell signaling in Helicobacter pylori infection](file:///D:\%E5%8D%8E%E5%A4%A7%E8%BD%AC%E5%BD%95%E7%BB%84%E6%95%B0%E6%8D%AE\GeneDiffExp\GeneDiffExp\Pathway\ACB-BtS-VS-ACB-AbR.html#gene39) | 16 (1.12%) | 146 (0.72%) | 0.05303007 | 3.208999e-01 | ko05120 |
| 40 | [Alzheimer's disease](file:///D:\%E5%8D%8E%E5%A4%A7%E8%BD%AC%E5%BD%95%E7%BB%84%E6%95%B0%E6%8D%AE\GeneDiffExp\GeneDiffExp\Pathway\ACB-BtS-VS-ACB-AbR.html#gene40) | 36 (2.53%) | 389 (1.93%) | 0.05869904 | 3.410504e-01 | ko05010 |
| 41 | [Olfactory transduction](file:///D:\%E5%8D%8E%E5%A4%A7%E8%BD%AC%E5%BD%95%E7%BB%84%E6%95%B0%E6%8D%AE\GeneDiffExp\GeneDiffExp\Pathway\ACB-BtS-VS-ACB-AbR.html#gene41) | 12 (0.84%) | 103 (0.51%) | 0.05925029 | 3.410504e-01 | ko04740 |
| 42 | [HTLV-I infection](file:///D:\%E5%8D%8E%E5%A4%A7%E8%BD%AC%E5%BD%95%E7%BB%84%E6%95%B0%E6%8D%AE\GeneDiffExp\GeneDiffExp\Pathway\ACB-BtS-VS-ACB-AbR.html#gene42) | 39 (2.74%) | 428 (2.12%) | 0.06144814 | 3.452800e-01 | ko05166 |
| 43 | [Butanoate metabolism](file:///D:\%E5%8D%8E%E5%A4%A7%E8%BD%AC%E5%BD%95%E7%BB%84%E6%95%B0%E6%8D%AE\GeneDiffExp\GeneDiffExp\Pathway\ACB-BtS-VS-ACB-AbR.html#gene43) | 12 (0.84%) | 105 (0.52%) | 0.06664903 | 3.657947e-01 | ko00650 |
| 44 | [Sphingolipid metabolism](file:///D:\%E5%8D%8E%E5%A4%A7%E8%BD%AC%E5%BD%95%E7%BB%84%E6%95%B0%E6%8D%AE\GeneDiffExp\GeneDiffExp\Pathway\ACB-BtS-VS-ACB-AbR.html#gene44) | 8 (0.56%) | 63 (0.31%) | 0.07475075 | 3.936743e-01 | ko00600 |
| 45 | [GABAergic synapse](file:///D:\%E5%8D%8E%E5%A4%A7%E8%BD%AC%E5%BD%95%E7%BB%84%E6%95%B0%E6%8D%AE\GeneDiffExp\GeneDiffExp\Pathway\ACB-BtS-VS-ACB-AbR.html#gene45) | 14 (0.98%) | 130 (0.65%) | 0.07522301 | 3.936743e-01 | ko04727 |
| 46 | [Antigen processing and presentation](file:///D:\%E5%8D%8E%E5%A4%A7%E8%BD%AC%E5%BD%95%E7%BB%84%E6%95%B0%E6%8D%AE\GeneDiffExp\GeneDiffExp\Pathway\ACB-BtS-VS-ACB-AbR.html#gene46) | 15 (1.05%) | 142 (0.7%) | 0.07673313 | 3.936743e-01 | ko04612 |
| 47 | [Mismatch repair](file:///D:\%E5%8D%8E%E5%A4%A7%E8%BD%AC%E5%BD%95%E7%BB%84%E6%95%B0%E6%8D%AE\GeneDiffExp\GeneDiffExp\Pathway\ACB-BtS-VS-ACB-AbR.html#gene47) | 8 (0.56%) | 65 (0.32%) | 0.08631933 | 4.295835e-01 | ko03430 |
| 48 | [Tuberculosis](file:///D:\%E5%8D%8E%E5%A4%A7%E8%BD%AC%E5%BD%95%E7%BB%84%E6%95%B0%E6%8D%AE\GeneDiffExp\GeneDiffExp\Pathway\ACB-BtS-VS-ACB-AbR.html#gene48) | 26 (1.83%) | 278 (1.38%) | 0.08737292 | 4.295835e-01 | ko05152 |
| 49 | [Ether lipid metabolism](file:///D:\%E5%8D%8E%E5%A4%A7%E8%BD%AC%E5%BD%95%E7%BB%84%E6%95%B0%E6%8D%AE\GeneDiffExp\GeneDiffExp\Pathway\ACB-BtS-VS-ACB-AbR.html#gene49) | 7 (0.49%) | 55 (0.27%) | 0.09111244 | 4.388273e-01 | ko00565 |
| 50 | [Renin-angiotensin system](file:///D:\%E5%8D%8E%E5%A4%A7%E8%BD%AC%E5%BD%95%E7%BB%84%E6%95%B0%E6%8D%AE\GeneDiffExp\GeneDiffExp\Pathway\ACB-BtS-VS-ACB-AbR.html#gene50) | 7 (0.49%) | 56 (0.28%) | 0.0981294 | 4.631708e-01 | ko04614 |
| 51 | [DNA replication](file:///D:\%E5%8D%8E%E5%A4%A7%E8%BD%AC%E5%BD%95%E7%BB%84%E6%95%B0%E6%8D%AE\GeneDiffExp\GeneDiffExp\Pathway\ACB-BtS-VS-ACB-AbR.html#gene51) | 12 (0.84%) | 114 (0.57%) | 0.1071842 | 4.959896e-01 | ko03030 |
| 52 | [Glycosphingolipid biosynthesis - globo series](file:///D:\%E5%8D%8E%E5%A4%A7%E8%BD%AC%E5%BD%95%E7%BB%84%E6%95%B0%E6%8D%AE\GeneDiffExp\GeneDiffExp\Pathway\ACB-BtS-VS-ACB-AbR.html#gene52) | 3 (0.21%) | 17 (0.08%) | 0.1140354 | 5.175453e-01 | ko00603 |
| 53 | [Primary bile acid biosynthesis](file:///D:\%E5%8D%8E%E5%A4%A7%E8%BD%AC%E5%BD%95%E7%BB%84%E6%95%B0%E6%8D%AE\GeneDiffExp\GeneDiffExp\Pathway\ACB-BtS-VS-ACB-AbR.html#gene53) | 4 (0.28%) | 27 (0.13%) | 0.1193553 | 5.277567e-01 | ko00120 |
| 54 | [Glycerophospholipid metabolism](file:///D:\%E5%8D%8E%E5%A4%A7%E8%BD%AC%E5%BD%95%E7%BB%84%E6%95%B0%E6%8D%AE\GeneDiffExp\GeneDiffExp\Pathway\ACB-BtS-VS-ACB-AbR.html#gene54) | 28 (1.97%) | 314 (1.56%) | 0.1207579 | 5.277567e-01 | ko00564 |
| 55 | [Cysteine and methionine metabolism](file:///D:\%E5%8D%8E%E5%A4%A7%E8%BD%AC%E5%BD%95%E7%BB%84%E6%95%B0%E6%8D%AE\GeneDiffExp\GeneDiffExp\Pathway\ACB-BtS-VS-ACB-AbR.html#gene55) | 8 (0.56%) | 74 (0.37%) | 0.1502869 | 6.448674e-01 | ko00270 |
| 56 | [Amyotrophic lateral sclerosis (ALS)](file:///D:\%E5%8D%8E%E5%A4%A7%E8%BD%AC%E5%BD%95%E7%BB%84%E6%95%B0%E6%8D%AE\GeneDiffExp\GeneDiffExp\Pathway\ACB-BtS-VS-ACB-AbR.html#gene56) | 17 (1.19%) | 185 (0.92%) | 0.160528 | 6.765109e-01 | ko05014 |
| 57 | [Terpenoid backbone biosynthesis](file:///D:\%E5%8D%8E%E5%A4%A7%E8%BD%AC%E5%BD%95%E7%BB%84%E6%95%B0%E6%8D%AE\GeneDiffExp\GeneDiffExp\Pathway\ACB-BtS-VS-ACB-AbR.html#gene57) | 6 (0.42%) | 54 (0.27%) | 0.1797405 | 7.441887e-01 | ko00900 |
| 58 | [Fatty acid elongation](file:///D:\%E5%8D%8E%E5%A4%A7%E8%BD%AC%E5%BD%95%E7%BB%84%E6%95%B0%E6%8D%AE\GeneDiffExp\GeneDiffExp\Pathway\ACB-BtS-VS-ACB-AbR.html#gene58) | 9 (0.63%) | 92 (0.46%) | 0.2011912 | 8.154108e-01 | ko00062 |
| 59 | [Nucleotide excision repair](file:///D:\%E5%8D%8E%E5%A4%A7%E8%BD%AC%E5%BD%95%E7%BB%84%E6%95%B0%E6%8D%AE\GeneDiffExp\GeneDiffExp\Pathway\ACB-BtS-VS-ACB-AbR.html#gene59) | 11 (0.77%) | 117 (0.58%) | 0.2038527 | 8.154108e-01 | ko03420 |
| 60 | [Pyrimidine metabolism](file:///D:\%E5%8D%8E%E5%A4%A7%E8%BD%AC%E5%BD%95%E7%BB%84%E6%95%B0%E6%8D%AE\GeneDiffExp\GeneDiffExp\Pathway\ACB-BtS-VS-ACB-AbR.html#gene60) | 39 (2.74%) | 483 (2.4%) | 0.2127501 | 8.368171e-01 | ko00240 |
| 61 | [Sulfur relay system](file:///D:\%E5%8D%8E%E5%A4%A7%E8%BD%AC%E5%BD%95%E7%BB%84%E6%95%B0%E6%8D%AE\GeneDiffExp\GeneDiffExp\Pathway\ACB-BtS-VS-ACB-AbR.html#gene61) | 4 (0.28%) | 34 (0.17%) | 0.2167555 | 8.385950e-01 | ko04122 |
| 62 | [Base excision repair](file:///D:\%E5%8D%8E%E5%A4%A7%E8%BD%AC%E5%BD%95%E7%BB%84%E6%95%B0%E6%8D%AE\GeneDiffExp\GeneDiffExp\Pathway\ACB-BtS-VS-ACB-AbR.html#gene62) | 7 (0.49%) | 71 (0.35%) | 0.2343596 | 8.920785e-01 | ko03410 |
| 63 | [Legionellosis](file:///D:\%E5%8D%8E%E5%A4%A7%E8%BD%AC%E5%BD%95%E7%BB%84%E6%95%B0%E6%8D%AE\GeneDiffExp\GeneDiffExp\Pathway\ACB-BtS-VS-ACB-AbR.html#gene63) | 15 (1.05%) | 180 (0.89%) | 0.2907323 | 1.000000e+00 | ko05134 |
| 64 | [Pyruvate metabolism](file:///D:\%E5%8D%8E%E5%A4%A7%E8%BD%AC%E5%BD%95%E7%BB%84%E6%95%B0%E6%8D%AE\GeneDiffExp\GeneDiffExp\Pathway\ACB-BtS-VS-ACB-AbR.html#gene64) | 13 (0.91%) | 155 (0.77%) | 0.3009768 | 1.000000e+00 | ko00620 |
| 65 | [Hematopoietic cell lineage](file:///D:\%E5%8D%8E%E5%A4%A7%E8%BD%AC%E5%BD%95%E7%BB%84%E6%95%B0%E6%8D%AE\GeneDiffExp\GeneDiffExp\Pathway\ACB-BtS-VS-ACB-AbR.html#gene65) | 9 (0.63%) | 105 (0.52%) | 0.3234416 | 1.000000e+00 | ko04640 |
| 66 | [Inositol phosphate metabolism](file:///D:\%E5%8D%8E%E5%A4%A7%E8%BD%AC%E5%BD%95%E7%BB%84%E6%95%B0%E6%8D%AE\GeneDiffExp\GeneDiffExp\Pathway\ACB-BtS-VS-ACB-AbR.html#gene66) | 11 (0.77%) | 132 (0.66%) | 0.3296835 | 1.000000e+00 | ko00562 |
| 67 | [Cardiac muscle contraction](file:///D:\%E5%8D%8E%E5%A4%A7%E8%BD%AC%E5%BD%95%E7%BB%84%E6%95%B0%E6%8D%AE\GeneDiffExp\GeneDiffExp\Pathway\ACB-BtS-VS-ACB-AbR.html#gene67) | 17 (1.19%) | 215 (1.07%) | 0.3505626 | 1.000000e+00 | ko04260 |
| 68 | [Citrate cycle (TCA cycle)](file:///D:\%E5%8D%8E%E5%A4%A7%E8%BD%AC%E5%BD%95%E7%BB%84%E6%95%B0%E6%8D%AE\GeneDiffExp\GeneDiffExp\Pathway\ACB-BtS-VS-ACB-AbR.html#gene68) | 9 (0.63%) | 108 (0.54%) | 0.353706 | 1.000000e+00 | ko00020 |
| 69 | [Measles](file:///D:\%E5%8D%8E%E5%A4%A7%E8%BD%AC%E5%BD%95%E7%BB%84%E6%95%B0%E6%8D%AE\GeneDiffExp\GeneDiffExp\Pathway\ACB-BtS-VS-ACB-AbR.html#gene69) | 18 (1.26%) | 229 (1.14%) | 0.3538191 | 1.000000e+00 | ko05162 |
| 70 | [Pancreatic secretion](file:///D:\%E5%8D%8E%E5%A4%A7%E8%BD%AC%E5%BD%95%E7%BB%84%E6%95%B0%E6%8D%AE\GeneDiffExp\GeneDiffExp\Pathway\ACB-BtS-VS-ACB-AbR.html#gene70) | 34 (2.39%) | 450 (2.23%) | 0.366484 | 1.000000e+00 | ko04972 |
| 71 | [Phototransduction](file:///D:\%E5%8D%8E%E5%A4%A7%E8%BD%AC%E5%BD%95%E7%BB%84%E6%95%B0%E6%8D%AE\GeneDiffExp\GeneDiffExp\Pathway\ACB-BtS-VS-ACB-AbR.html#gene71) | 7 (0.49%) | 83 (0.41%) | 0.3707778 | 1.000000e+00 | ko04744 |
| 72 | [One carbon pool by folate](file:///D:\%E5%8D%8E%E5%A4%A7%E8%BD%AC%E5%BD%95%E7%BB%84%E6%95%B0%E6%8D%AE\GeneDiffExp\GeneDiffExp\Pathway\ACB-BtS-VS-ACB-AbR.html#gene72) | 3 (0.21%) | 31 (0.15%) | 0.3763883 | 1.000000e+00 | ko00670 |
| 73 | [Retrograde endocannabinoid signaling](file:///D:\%E5%8D%8E%E5%A4%A7%E8%BD%AC%E5%BD%95%E7%BB%84%E6%95%B0%E6%8D%AE\GeneDiffExp\GeneDiffExp\Pathway\ACB-BtS-VS-ACB-AbR.html#gene73) | 11 (0.77%) | 138 (0.69%) | 0.3841588 | 1.000000e+00 | ko04723 |
| 74 | [B cell receptor signaling pathway](file:///D:\%E5%8D%8E%E5%A4%A7%E8%BD%AC%E5%BD%95%E7%BB%84%E6%95%B0%E6%8D%AE\GeneDiffExp\GeneDiffExp\Pathway\ACB-BtS-VS-ACB-AbR.html#gene74) | 8 (0.56%) | 98 (0.49%) | 0.389241 | 1.000000e+00 | ko04662 |
| 75 | [Linoleic acid metabolism](file:///D:\%E5%8D%8E%E5%A4%A7%E8%BD%AC%E5%BD%95%E7%BB%84%E6%95%B0%E6%8D%AE\GeneDiffExp\GeneDiffExp\Pathway\ACB-BtS-VS-ACB-AbR.html#gene75) | 7 (0.49%) | 85 (0.42%) | 0.3943546 | 1.000000e+00 | ko00591 |
| 76 | [Spliceosome](file:///D:\%E5%8D%8E%E5%A4%A7%E8%BD%AC%E5%BD%95%E7%BB%84%E6%95%B0%E6%8D%AE\GeneDiffExp\GeneDiffExp\Pathway\ACB-BtS-VS-ACB-AbR.html#gene76) | 54 (3.79%) | 735 (3.65%) | 0.4013455 | 1.000000e+00 | ko03040 |
| 77 | [Type I diabetes mellitus](file:///D:\%E5%8D%8E%E5%A4%A7%E8%BD%AC%E5%BD%95%E7%BB%84%E6%95%B0%E6%8D%AE\GeneDiffExp\GeneDiffExp\Pathway\ACB-BtS-VS-ACB-AbR.html#gene77) | 2 (0.14%) | 20 (0.1%) | 0.4178174 | 1.000000e+00 | ko04940 |
| 78 | [Amphetamine addiction](file:///D:\%E5%8D%8E%E5%A4%A7%E8%BD%AC%E5%BD%95%E7%BB%84%E6%95%B0%E6%8D%AE\GeneDiffExp\GeneDiffExp\Pathway\ACB-BtS-VS-ACB-AbR.html#gene78) | 10 (0.7%) | 128 (0.64%) | 0.4182735 | 1.000000e+00 | ko05031 |
| 79 | [Morphine addiction](file:///D:\%E5%8D%8E%E5%A4%A7%E8%BD%AC%E5%BD%95%E7%BB%84%E6%95%B0%E6%8D%AE\GeneDiffExp\GeneDiffExp\Pathway\ACB-BtS-VS-ACB-AbR.html#gene79) | 10 (0.7%) | 129 (0.64%) | 0.4279559 | 1.000000e+00 | ko05032 |
| 80 | [TGF-beta signaling pathway](file:///D:\%E5%8D%8E%E5%A4%A7%E8%BD%AC%E5%BD%95%E7%BB%84%E6%95%B0%E6%8D%AE\GeneDiffExp\GeneDiffExp\Pathway\ACB-BtS-VS-ACB-AbR.html#gene80) | 10 (0.7%) | 129 (0.64%) | 0.4279559 | 1.000000e+00 | ko04350 |
| 81 | [p53 signaling pathway](file:///D:\%E5%8D%8E%E5%A4%A7%E8%BD%AC%E5%BD%95%E7%BB%84%E6%95%B0%E6%8D%AE\GeneDiffExp\GeneDiffExp\Pathway\ACB-BtS-VS-ACB-AbR.html#gene81) | 9 (0.63%) | 116 (0.58%) | 0.4355677 | 1.000000e+00 | ko04115 |
| 82 | [Non-homologous end-joining](file:///D:\%E5%8D%8E%E5%A4%A7%E8%BD%AC%E5%BD%95%E7%BB%84%E6%95%B0%E6%8D%AE\GeneDiffExp\GeneDiffExp\Pathway\ACB-BtS-VS-ACB-AbR.html#gene82) | 2 (0.14%) | 21 (0.1%) | 0.4426423 | 1.000000e+00 | ko03450 |
| 83 | [Colorectal cancer](file:///D:\%E5%8D%8E%E5%A4%A7%E8%BD%AC%E5%BD%95%E7%BB%84%E6%95%B0%E6%8D%AE\GeneDiffExp\GeneDiffExp\Pathway\ACB-BtS-VS-ACB-AbR.html#gene83) | 8 (0.56%) | 103 (0.51%) | 0.4438902 | 1.000000e+00 | ko05210 |
| 84 | [Alanine, aspartate and glutamate metabolism](file:///D:\%E5%8D%8E%E5%A4%A7%E8%BD%AC%E5%BD%95%E7%BB%84%E6%95%B0%E6%8D%AE\GeneDiffExp\GeneDiffExp\Pathway\ACB-BtS-VS-ACB-AbR.html#gene84) | 8 (0.56%) | 104 (0.52%) | 0.454759 | 1.000000e+00 | ko00250 |
| 85 | [Glycine, serine and threonine metabolism](file:///D:\%E5%8D%8E%E5%A4%A7%E8%BD%AC%E5%BD%95%E7%BB%84%E6%95%B0%E6%8D%AE\GeneDiffExp\GeneDiffExp\Pathway\ACB-BtS-VS-ACB-AbR.html#gene85) | 12 (0.84%) | 160 (0.79%) | 0.4582142 | 1.000000e+00 | ko00260 |
| 86 | [Metabolic pathways](file:///D:\%E5%8D%8E%E5%A4%A7%E8%BD%AC%E5%BD%95%E7%BB%84%E6%95%B0%E6%8D%AE\GeneDiffExp\GeneDiffExp\Pathway\ACB-BtS-VS-ACB-AbR.html#gene86) | 250 (17.57%) | 3514 (17.44%) | 0.460792 | 1.000000e+00 | ko01100 |
| 87 | [Valine, leucine and isoleucine degradation](file:///D:\%E5%8D%8E%E5%A4%A7%E8%BD%AC%E5%BD%95%E7%BB%84%E6%95%B0%E6%8D%AE\GeneDiffExp\GeneDiffExp\Pathway\ACB-BtS-VS-ACB-AbR.html#gene87) | 11 (0.77%) | 147 (0.73%) | 0.4667966 | 1.000000e+00 | ko00280 |
| 88 | [Endocrine and other factor-regulated calcium reabsorption](file:///D:\%E5%8D%8E%E5%A4%A7%E8%BD%AC%E5%BD%95%E7%BB%84%E6%95%B0%E6%8D%AE\GeneDiffExp\GeneDiffExp\Pathway\ACB-BtS-VS-ACB-AbR.html#gene88) | 8 (0.56%) | 107 (0.53%) | 0.4871055 | 1.000000e+00 | ko04961 |
| 89 | [Natural killer cell mediated cytotoxicity](file:///D:\%E5%8D%8E%E5%A4%A7%E8%BD%AC%E5%BD%95%E7%BB%84%E6%95%B0%E6%8D%AE\GeneDiffExp\GeneDiffExp\Pathway\ACB-BtS-VS-ACB-AbR.html#gene89) | 7 (0.49%) | 94 (0.47%) | 0.4993035 | 1.000000e+00 | ko04650 |
| 90 | [Cytosolic DNA-sensing pathway](file:///D:\%E5%8D%8E%E5%A4%A7%E8%BD%AC%E5%BD%95%E7%BB%84%E6%95%B0%E6%8D%AE\GeneDiffExp\GeneDiffExp\Pathway\ACB-BtS-VS-ACB-AbR.html#gene90) | 11 (0.77%) | 151 (0.75%) | 0.5030726 | 1.000000e+00 | ko04623 |
| 91 | [RNA transport](file:///D:\%E5%8D%8E%E5%A4%A7%E8%BD%AC%E5%BD%95%E7%BB%84%E6%95%B0%E6%8D%AE\GeneDiffExp\GeneDiffExp\Pathway\ACB-BtS-VS-ACB-AbR.html#gene91) | 55 (3.87%) | 780 (3.87%) | 0.5266253 | 1.000000e+00 | ko03013 |
| 92 | [Type II diabetes mellitus](file:///D:\%E5%8D%8E%E5%A4%A7%E8%BD%AC%E5%BD%95%E7%BB%84%E6%95%B0%E6%8D%AE\GeneDiffExp\GeneDiffExp\Pathway\ACB-BtS-VS-ACB-AbR.html#gene92) | 7 (0.49%) | 98 (0.49%) | 0.5441679 | 1.000000e+00 | ko04930 |
| 93 | [Biotin metabolism](file:///D:\%E5%8D%8E%E5%A4%A7%E8%BD%AC%E5%BD%95%E7%BB%84%E6%95%B0%E6%8D%AE\GeneDiffExp\GeneDiffExp\Pathway\ACB-BtS-VS-ACB-AbR.html#gene93) | 1 (0.07%) | 11 (0.05%) | 0.553392 | 1.000000e+00 | ko00780 |
| 94 | [mTOR signaling pathway](file:///D:\%E5%8D%8E%E5%A4%A7%E8%BD%AC%E5%BD%95%E7%BB%84%E6%95%B0%E6%8D%AE\GeneDiffExp\GeneDiffExp\Pathway\ACB-BtS-VS-ACB-AbR.html#gene94) | 9 (0.63%) | 128 (0.64%) | 0.5553337 | 1.000000e+00 | ko04150 |
| 95 | [Arachidonic acid metabolism](file:///D:\%E5%8D%8E%E5%A4%A7%E8%BD%AC%E5%BD%95%E7%BB%84%E6%95%B0%E6%8D%AE\GeneDiffExp\GeneDiffExp\Pathway\ACB-BtS-VS-ACB-AbR.html#gene95) | 5 (0.35%) | 71 (0.35%) | 0.5690636 | 1.000000e+00 | ko00590 |
| 96 | [Autoimmune thyroid disease](file:///D:\%E5%8D%8E%E5%A4%A7%E8%BD%AC%E5%BD%95%E7%BB%84%E6%95%B0%E6%8D%AE\GeneDiffExp\GeneDiffExp\Pathway\ACB-BtS-VS-ACB-AbR.html#gene96) | 2 (0.14%) | 27 (0.13%) | 0.5779086 | 1.000000e+00 | ko05320 |
| 97 | [Osteoclast differentiation](file:///D:\%E5%8D%8E%E5%A4%A7%E8%BD%AC%E5%BD%95%E7%BB%84%E6%95%B0%E6%8D%AE\GeneDiffExp\GeneDiffExp\Pathway\ACB-BtS-VS-ACB-AbR.html#gene97) | 8 (0.56%) | 120 (0.6%) | 0.6190513 | 1.000000e+00 | ko04380 |
| 98 | [Adherens junction](file:///D:\%E5%8D%8E%E5%A4%A7%E8%BD%AC%E5%BD%95%E7%BB%84%E6%95%B0%E6%8D%AE\GeneDiffExp\GeneDiffExp\Pathway\ACB-BtS-VS-ACB-AbR.html#gene98) | 19 (1.34%) | 284 (1.41%) | 0.6314229 | 1.000000e+00 | ko04520 |
| 99 | [Prion diseases](file:///D:\%E5%8D%8E%E5%A4%A7%E8%BD%AC%E5%BD%95%E7%BB%84%E6%95%B0%E6%8D%AE\GeneDiffExp\GeneDiffExp\Pathway\ACB-BtS-VS-ACB-AbR.html#gene99) | 12 (0.84%) | 181 (0.9%) | 0.6325148 | 1.000000e+00 | ko05020 |
| 100 | [Folate biosynthesis](file:///D:\%E5%8D%8E%E5%A4%A7%E8%BD%AC%E5%BD%95%E7%BB%84%E6%95%B0%E6%8D%AE\GeneDiffExp\GeneDiffExp\Pathway\ACB-BtS-VS-ACB-AbR.html#gene100) | 4 (0.28%) | 61 (0.3%) | 0.6334537 | 1.000000e+00 | ko00790 |
| 101 | [Selenocompound metabolism](file:///D:\%E5%8D%8E%E5%A4%A7%E8%BD%AC%E5%BD%95%E7%BB%84%E6%95%B0%E6%8D%AE\GeneDiffExp\GeneDiffExp\Pathway\ACB-BtS-VS-ACB-AbR.html#gene101) | 2 (0.14%) | 31 (0.15%) | 0.6538413 | 1.000000e+00 | ko00450 |
| 102 | [Endocytosis](file:///D:\%E5%8D%8E%E5%A4%A7%E8%BD%AC%E5%BD%95%E7%BB%84%E6%95%B0%E6%8D%AE\GeneDiffExp\GeneDiffExp\Pathway\ACB-BtS-VS-ACB-AbR.html#gene102) | 34 (2.39%) | 508 (2.52%) | 0.65486 | 1.000000e+00 | ko04144 |
| 103 | [Wnt signaling pathway](file:///D:\%E5%8D%8E%E5%A4%A7%E8%BD%AC%E5%BD%95%E7%BB%84%E6%95%B0%E6%8D%AE\GeneDiffExp\GeneDiffExp\Pathway\ACB-BtS-VS-ACB-AbR.html#gene103) | 19 (1.34%) | 289 (1.43%) | 0.6614729 | 1.000000e+00 | ko04310 |
| 104 | [NOD-like receptor signaling pathway](file:///D:\%E5%8D%8E%E5%A4%A7%E8%BD%AC%E5%BD%95%E7%BB%84%E6%95%B0%E6%8D%AE\GeneDiffExp\GeneDiffExp\Pathway\ACB-BtS-VS-ACB-AbR.html#gene104) | 6 (0.42%) | 95 (0.47%) | 0.6700989 | 1.000000e+00 | ko04621 |
| 105 | [Circadian rhythm - fly](file:///D:\%E5%8D%8E%E5%A4%A7%E8%BD%AC%E5%BD%95%E7%BB%84%E6%95%B0%E6%8D%AE\GeneDiffExp\GeneDiffExp\Pathway\ACB-BtS-VS-ACB-AbR.html#gene105) | 2 (0.14%) | 32 (0.16%) | 0.6710314 | 1.000000e+00 | ko04711 |
| 106 | [Bacterial invasion of epithelial cells](file:///D:\%E5%8D%8E%E5%A4%A7%E8%BD%AC%E5%BD%95%E7%BB%84%E6%95%B0%E6%8D%AE\GeneDiffExp\GeneDiffExp\Pathway\ACB-BtS-VS-ACB-AbR.html#gene106) | 17 (1.19%) | 262 (1.3%) | 0.6775716 | 1.000000e+00 | ko05100 |
| 107 | [ECM-receptor interaction](file:///D:\%E5%8D%8E%E5%A4%A7%E8%BD%AC%E5%BD%95%E7%BB%84%E6%95%B0%E6%8D%AE\GeneDiffExp\GeneDiffExp\Pathway\ACB-BtS-VS-ACB-AbR.html#gene107) | 20 (1.41%) | 308 (1.53%) | 0.6851969 | 1.000000e+00 | ko04512 |
| 108 | [Butirosin and neomycin biosynthesis](file:///D:\%E5%8D%8E%E5%A4%A7%E8%BD%AC%E5%BD%95%E7%BB%84%E6%95%B0%E6%8D%AE\GeneDiffExp\GeneDiffExp\Pathway\ACB-BtS-VS-ACB-AbR.html#gene108) | 1 (0.07%) | 16 (0.08%) | 0.6904459 | 1.000000e+00 | ko00524 |
| 109 | [Adipocytokine signaling pathway](file:///D:\%E5%8D%8E%E5%A4%A7%E8%BD%AC%E5%BD%95%E7%BB%84%E6%95%B0%E6%8D%AE\GeneDiffExp\GeneDiffExp\Pathway\ACB-BtS-VS-ACB-AbR.html#gene109) | 7 (0.49%) | 113 (0.56%) | 0.6940945 | 1.000000e+00 | ko04920 |
| 110 | [SNARE interactions in vesicular transport](file:///D:\%E5%8D%8E%E5%A4%A7%E8%BD%AC%E5%BD%95%E7%BB%84%E6%95%B0%E6%8D%AE\GeneDiffExp\GeneDiffExp\Pathway\ACB-BtS-VS-ACB-AbR.html#gene110) | 4 (0.28%) | 66 (0.33%) | 0.6944942 | 1.000000e+00 | ko04130 |
| 111 | [Taurine and hypotaurine metabolism](file:///D:\%E5%8D%8E%E5%A4%A7%E8%BD%AC%E5%BD%95%E7%BB%84%E6%95%B0%E6%8D%AE\GeneDiffExp\GeneDiffExp\Pathway\ACB-BtS-VS-ACB-AbR.html#gene111) | 1 (0.07%) | 17 (0.08%) | 0.7123306 | 1.000000e+00 | ko00430 |
| 112 | [Acute myeloid leukemia](file:///D:\%E5%8D%8E%E5%A4%A7%E8%BD%AC%E5%BD%95%E7%BB%84%E6%95%B0%E6%8D%AE\GeneDiffExp\GeneDiffExp\Pathway\ACB-BtS-VS-ACB-AbR.html#gene112) | 5 (0.35%) | 84 (0.42%) | 0.7162358 | 1.000000e+00 | ko05221 |
| 113 | [RNA polymerase](file:///D:\%E5%8D%8E%E5%A4%A7%E8%BD%AC%E5%BD%95%E7%BB%84%E6%95%B0%E6%8D%AE\GeneDiffExp\GeneDiffExp\Pathway\ACB-BtS-VS-ACB-AbR.html#gene113) | 21 (1.48%) | 329 (1.63%) | 0.717313 | 1.000000e+00 | ko03020 |
| 114 | [Renal cell carcinoma](file:///D:\%E5%8D%8E%E5%A4%A7%E8%BD%AC%E5%BD%95%E7%BB%84%E6%95%B0%E6%8D%AE\GeneDiffExp\GeneDiffExp\Pathway\ACB-BtS-VS-ACB-AbR.html#gene114) | 9 (0.63%) | 147 (0.73%) | 0.7187307 | 1.000000e+00 | ko05211 |
| 115 | [Long-term depression](file:///D:\%E5%8D%8E%E5%A4%A7%E8%BD%AC%E5%BD%95%E7%BB%84%E6%95%B0%E6%8D%AE\GeneDiffExp\GeneDiffExp\Pathway\ACB-BtS-VS-ACB-AbR.html#gene115) | 5 (0.35%) | 85 (0.42%) | 0.725919 | 1.000000e+00 | ko04730 |
| 116 | [Progesterone-mediated oocyte maturation](file:///D:\%E5%8D%8E%E5%A4%A7%E8%BD%AC%E5%BD%95%E7%BB%84%E6%95%B0%E6%8D%AE\GeneDiffExp\GeneDiffExp\Pathway\ACB-BtS-VS-ACB-AbR.html#gene116) | 18 (1.26%) | 286 (1.42%) | 0.7285528 | 1.000000e+00 | ko04914 |
| 117 | [Alcoholism](file:///D:\%E5%8D%8E%E5%A4%A7%E8%BD%AC%E5%BD%95%E7%BB%84%E6%95%B0%E6%8D%AE\GeneDiffExp\GeneDiffExp\Pathway\ACB-BtS-VS-ACB-AbR.html#gene117) | 12 (0.84%) | 195 (0.97%) | 0.7307347 | 1.000000e+00 | ko05034 |
| 118 | [Glycosphingolipid biosynthesis - lacto and neolacto series](file:///D:\%E5%8D%8E%E5%A4%A7%E8%BD%AC%E5%BD%95%E7%BB%84%E6%95%B0%E6%8D%AE\GeneDiffExp\GeneDiffExp\Pathway\ACB-BtS-VS-ACB-AbR.html#gene118) | 1 (0.07%) | 18 (0.09%) | 0.7326692 | 1.000000e+00 | ko00601 |
| 119 | [Ubiquitin mediated proteolysis](file:///D:\%E5%8D%8E%E5%A4%A7%E8%BD%AC%E5%BD%95%E7%BB%84%E6%95%B0%E6%8D%AE\GeneDiffExp\GeneDiffExp\Pathway\ACB-BtS-VS-ACB-AbR.html#gene119) | 31 (2.18%) | 482 (2.39%) | 0.7336594 | 1.000000e+00 | ko04120 |
| 120 | [Toxoplasmosis](file:///D:\%E5%8D%8E%E5%A4%A7%E8%BD%AC%E5%BD%95%E7%BB%84%E6%95%B0%E6%8D%AE\GeneDiffExp\GeneDiffExp\Pathway\ACB-BtS-VS-ACB-AbR.html#gene120) | 18 (1.26%) | 288 (1.43%) | 0.7390536 | 1.000000e+00 | ko05145 |
| 121 | [Tight junction](file:///D:\%E5%8D%8E%E5%A4%A7%E8%BD%AC%E5%BD%95%E7%BB%84%E6%95%B0%E6%8D%AE\GeneDiffExp\GeneDiffExp\Pathway\ACB-BtS-VS-ACB-AbR.html#gene121) | 22 (1.55%) | 351 (1.74%) | 0.7508723 | 1.000000e+00 | ko04530 |
| 122 | [African trypanosomiasis](file:///D:\%E5%8D%8E%E5%A4%A7%E8%BD%AC%E5%BD%95%E7%BB%84%E6%95%B0%E6%8D%AE\GeneDiffExp\GeneDiffExp\Pathway\ACB-BtS-VS-ACB-AbR.html#gene122) | 1 (0.07%) | 19 (0.09%) | 0.7515707 | 1.000000e+00 | ko05143 |
| 123 | [Notch signaling pathway](file:///D:\%E5%8D%8E%E5%A4%A7%E8%BD%AC%E5%BD%95%E7%BB%84%E6%95%B0%E6%8D%AE\GeneDiffExp\GeneDiffExp\Pathway\ACB-BtS-VS-ACB-AbR.html#gene123) | 7 (0.49%) | 124 (0.62%) | 0.7811863 | 1.000000e+00 | ko04330 |
| 124 | [Tyrosine metabolism](file:///D:\%E5%8D%8E%E5%A4%A7%E8%BD%AC%E5%BD%95%E7%BB%84%E6%95%B0%E6%8D%AE\GeneDiffExp\GeneDiffExp\Pathway\ACB-BtS-VS-ACB-AbR.html#gene124) | 9 (0.63%) | 157 (0.78%) | 0.7867887 | 1.000000e+00 | ko00350 |
| 125 | [NF-kappa B signaling pathway](file:///D:\%E5%8D%8E%E5%A4%A7%E8%BD%AC%E5%BD%95%E7%BB%84%E6%95%B0%E6%8D%AE\GeneDiffExp\GeneDiffExp\Pathway\ACB-BtS-VS-ACB-AbR.html#gene125) | 6 (0.42%) | 109 (0.54%) | 0.7905005 | 1.000000e+00 | ko04064 |
| 126 | [RIG-I-like receptor signaling pathway](file:///D:\%E5%8D%8E%E5%A4%A7%E8%BD%AC%E5%BD%95%E7%BB%84%E6%95%B0%E6%8D%AE\GeneDiffExp\GeneDiffExp\Pathway\ACB-BtS-VS-ACB-AbR.html#gene126) | 3 (0.21%) | 59 (0.29%) | 0.796482 | 1.000000e+00 | ko04622 |
| 127 | [Glyoxylate and dicarboxylate metabolism](file:///D:\%E5%8D%8E%E5%A4%A7%E8%BD%AC%E5%BD%95%E7%BB%84%E6%95%B0%E6%8D%AE\GeneDiffExp\GeneDiffExp\Pathway\ACB-BtS-VS-ACB-AbR.html#gene127) | 3 (0.21%) | 59 (0.29%) | 0.796482 | 1.000000e+00 | ko00630 |
| 128 | [Hepatitis C](file:///D:\%E5%8D%8E%E5%A4%A7%E8%BD%AC%E5%BD%95%E7%BB%84%E6%95%B0%E6%8D%AE\GeneDiffExp\GeneDiffExp\Pathway\ACB-BtS-VS-ACB-AbR.html#gene128) | 8 (0.56%) | 145 (0.72%) | 0.8117178 | 1.000000e+00 | ko05160 |
| 129 | [Long-term potentiation](file:///D:\%E5%8D%8E%E5%A4%A7%E8%BD%AC%E5%BD%95%E7%BB%84%E6%95%B0%E6%8D%AE\GeneDiffExp\GeneDiffExp\Pathway\ACB-BtS-VS-ACB-AbR.html#gene129) | 8 (0.56%) | 145 (0.72%) | 0.8117178 | 1.000000e+00 | ko04720 |
| 130 | [Salivary secretion](file:///D:\%E5%8D%8E%E5%A4%A7%E8%BD%AC%E5%BD%95%E7%BB%84%E6%95%B0%E6%8D%AE\GeneDiffExp\GeneDiffExp\Pathway\ACB-BtS-VS-ACB-AbR.html#gene130) | 18 (1.26%) | 304 (1.51%) | 0.813573 | 1.000000e+00 | ko04970 |
| 131 | [Phosphatidylinositol signaling system](file:///D:\%E5%8D%8E%E5%A4%A7%E8%BD%AC%E5%BD%95%E7%BB%84%E6%95%B0%E6%8D%AE\GeneDiffExp\GeneDiffExp\Pathway\ACB-BtS-VS-ACB-AbR.html#gene131) | 11 (0.77%) | 194 (0.96%) | 0.8148898 | 1.000000e+00 | ko04070 |
| 132 | [ErbB signaling pathway](file:///D:\%E5%8D%8E%E5%A4%A7%E8%BD%AC%E5%BD%95%E7%BB%84%E6%95%B0%E6%8D%AE\GeneDiffExp\GeneDiffExp\Pathway\ACB-BtS-VS-ACB-AbR.html#gene132) | 8 (0.56%) | 146 (0.72%) | 0.8175445 | 1.000000e+00 | ko04012 |
| 133 | [alpha-Linolenic acid metabolism](file:///D:\%E5%8D%8E%E5%A4%A7%E8%BD%AC%E5%BD%95%E7%BB%84%E6%95%B0%E6%8D%AE\GeneDiffExp\GeneDiffExp\Pathway\ACB-BtS-VS-ACB-AbR.html#gene133) | 7 (0.49%) | 131 (0.65%) | 0.8261384 | 1.000000e+00 | ko00592 |
| 134 | [VEGF signaling pathway](file:///D:\%E5%8D%8E%E5%A4%A7%E8%BD%AC%E5%BD%95%E7%BB%84%E6%95%B0%E6%8D%AE\GeneDiffExp\GeneDiffExp\Pathway\ACB-BtS-VS-ACB-AbR.html#gene134) | 8 (0.56%) | 148 (0.73%) | 0.8287806 | 1.000000e+00 | ko04370 |
| 135 | [Dopaminergic synapse](file:///D:\%E5%8D%8E%E5%A4%A7%E8%BD%AC%E5%BD%95%E7%BB%84%E6%95%B0%E6%8D%AE\GeneDiffExp\GeneDiffExp\Pathway\ACB-BtS-VS-ACB-AbR.html#gene135) | 11 (0.77%) | 199 (0.99%) | 0.8390189 | 1.000000e+00 | ko04728 |
| 136 | [Cyanoamino acid metabolism](file:///D:\%E5%8D%8E%E5%A4%A7%E8%BD%AC%E5%BD%95%E7%BB%84%E6%95%B0%E6%8D%AE\GeneDiffExp\GeneDiffExp\Pathway\ACB-BtS-VS-ACB-AbR.html#gene136) | 1 (0.07%) | 25 (0.12%) | 0.8400106 | 1.000000e+00 | ko00460 |
| 137 | [Lysine degradation](file:///D:\%E5%8D%8E%E5%A4%A7%E8%BD%AC%E5%BD%95%E7%BB%84%E6%95%B0%E6%8D%AE\GeneDiffExp\GeneDiffExp\Pathway\ACB-BtS-VS-ACB-AbR.html#gene137) | 14 (0.98%) | 248 (1.23%) | 0.8423606 | 1.000000e+00 | ko00310 |
| 138 | [Jak-STAT signaling pathway](file:///D:\%E5%8D%8E%E5%A4%A7%E8%BD%AC%E5%BD%95%E7%BB%84%E6%95%B0%E6%8D%AE\GeneDiffExp\GeneDiffExp\Pathway\ACB-BtS-VS-ACB-AbR.html#gene138) | 6 (0.42%) | 117 (0.58%) | 0.8424166 | 1.000000e+00 | ko04630 |
| 139 | [Tryptophan metabolism](file:///D:\%E5%8D%8E%E5%A4%A7%E8%BD%AC%E5%BD%95%E7%BB%84%E6%95%B0%E6%8D%AE\GeneDiffExp\GeneDiffExp\Pathway\ACB-BtS-VS-ACB-AbR.html#gene139) | 6 (0.42%) | 118 (0.59%) | 0.8481061 | 1.000000e+00 | ko00380 |
| 140 | [Sulfur metabolism](file:///D:\%E5%8D%8E%E5%A4%A7%E8%BD%AC%E5%BD%95%E7%BB%84%E6%95%B0%E6%8D%AE\GeneDiffExp\GeneDiffExp\Pathway\ACB-BtS-VS-ACB-AbR.html#gene140) | 1 (0.07%) | 26 (0.13%) | 0.8513265 | 1.000000e+00 | ko00920 |
| 141 | [Synthesis and degradation of ketone bodies](file:///D:\%E5%8D%8E%E5%A4%A7%E8%BD%AC%E5%BD%95%E7%BB%84%E6%95%B0%E6%8D%AE\GeneDiffExp\GeneDiffExp\Pathway\ACB-BtS-VS-ACB-AbR.html#gene141) | 1 (0.07%) | 26 (0.13%) | 0.8513265 | 1.000000e+00 | ko00072 |
| 142 | [Apoptosis](file:///D:\%E5%8D%8E%E5%A4%A7%E8%BD%AC%E5%BD%95%E7%BB%84%E6%95%B0%E6%8D%AE\GeneDiffExp\GeneDiffExp\Pathway\ACB-BtS-VS-ACB-AbR.html#gene142) | 5 (0.35%) | 102 (0.51%) | 0.855265 | 1.000000e+00 | ko04210 |
| 143 | [Fatty acid metabolism](file:///D:\%E5%8D%8E%E5%A4%A7%E8%BD%AC%E5%BD%95%E7%BB%84%E6%95%B0%E6%8D%AE\GeneDiffExp\GeneDiffExp\Pathway\ACB-BtS-VS-ACB-AbR.html#gene143) | 7 (0.49%) | 137 (0.68%) | 0.8585976 | 1.000000e+00 | ko00071 |
| 144 | [Galactose metabolism](file:///D:\%E5%8D%8E%E5%A4%A7%E8%BD%AC%E5%BD%95%E7%BB%84%E6%95%B0%E6%8D%AE\GeneDiffExp\GeneDiffExp\Pathway\ACB-BtS-VS-ACB-AbR.html#gene144) | 20 (1.41%) | 348 (1.73%) | 0.8597074 | 1.000000e+00 | ko00052 |
| 145 | [Toll-like receptor signaling pathway](file:///D:\%E5%8D%8E%E5%A4%A7%E8%BD%AC%E5%BD%95%E7%BB%84%E6%95%B0%E6%8D%AE\GeneDiffExp\GeneDiffExp\Pathway\ACB-BtS-VS-ACB-AbR.html#gene145) | 5 (0.35%) | 103 (0.51%) | 0.8609607 | 1.000000e+00 | ko04620 |
| 146 | [Leukocyte transendothelial migration](file:///D:\%E5%8D%8E%E5%A4%A7%E8%BD%AC%E5%BD%95%E7%BB%84%E6%95%B0%E6%8D%AE\GeneDiffExp\GeneDiffExp\Pathway\ACB-BtS-VS-ACB-AbR.html#gene146) | 12 (0.84%) | 222 (1.1%) | 0.8671135 | 1.000000e+00 | ko04670 |
| 147 | [Hypertrophic cardiomyopathy (HCM)](file:///D:\%E5%8D%8E%E5%A4%A7%E8%BD%AC%E5%BD%95%E7%BB%84%E6%95%B0%E6%8D%AE\GeneDiffExp\GeneDiffExp\Pathway\ACB-BtS-VS-ACB-AbR.html#gene147) | 18 (1.26%) | 319 (1.58%) | 0.8682859 | 1.000000e+00 | ko05410 |
| 148 | [Complement and coagulation cascades](file:///D:\%E5%8D%8E%E5%A4%A7%E8%BD%AC%E5%BD%95%E7%BB%84%E6%95%B0%E6%8D%AE\GeneDiffExp\GeneDiffExp\Pathway\ACB-BtS-VS-ACB-AbR.html#gene148) | 5 (0.35%) | 105 (0.52%) | 0.8717887 | 1.000000e+00 | ko04610 |
| 149 | [Hedgehog signaling pathway](file:///D:\%E5%8D%8E%E5%A4%A7%E8%BD%AC%E5%BD%95%E7%BB%84%E6%95%B0%E6%8D%AE\GeneDiffExp\GeneDiffExp\Pathway\ACB-BtS-VS-ACB-AbR.html#gene149) | 5 (0.35%) | 107 (0.53%) | 0.881894 | 1.000000e+00 | ko04340 |
| 150 | [Starch and sucrose metabolism](file:///D:\%E5%8D%8E%E5%A4%A7%E8%BD%AC%E5%BD%95%E7%BB%84%E6%95%B0%E6%8D%AE\GeneDiffExp\GeneDiffExp\Pathway\ACB-BtS-VS-ACB-AbR.html#gene150) | 21 (1.48%) | 372 (1.85%) | 0.8837552 | 1.000000e+00 | ko00500 |
| 151 | [Porphyrin and chlorophyll metabolism](file:///D:\%E5%8D%8E%E5%A4%A7%E8%BD%AC%E5%BD%95%E7%BB%84%E6%95%B0%E6%8D%AE\GeneDiffExp\GeneDiffExp\Pathway\ACB-BtS-VS-ACB-AbR.html#gene151) | 4 (0.28%) | 90 (0.45%) | 0.8874718 | 1.000000e+00 | ko00860 |
| 152 | [Small cell lung cancer](file:///D:\%E5%8D%8E%E5%A4%A7%E8%BD%AC%E5%BD%95%E7%BB%84%E6%95%B0%E6%8D%AE\GeneDiffExp\GeneDiffExp\Pathway\ACB-BtS-VS-ACB-AbR.html#gene152) | 9 (0.63%) | 178 (0.88%) | 0.8890101 | 1.000000e+00 | ko05222 |
| 153 | [Pathways in cancer](file:///D:\%E5%8D%8E%E5%A4%A7%E8%BD%AC%E5%BD%95%E7%BB%84%E6%95%B0%E6%8D%AE\GeneDiffExp\GeneDiffExp\Pathway\ACB-BtS-VS-ACB-AbR.html#gene153) | 34 (2.39%) | 580 (2.88%) | 0.8928695 | 1.000000e+00 | ko05200 |
| 154 | [Pentose and glucuronate interconversions](file:///D:\%E5%8D%8E%E5%A4%A7%E8%BD%AC%E5%BD%95%E7%BB%84%E6%95%B0%E6%8D%AE\GeneDiffExp\GeneDiffExp\Pathway\ACB-BtS-VS-ACB-AbR.html#gene154) | 10 (0.7%) | 196 (0.97%) | 0.8929651 | 1.000000e+00 | ko00040 |
| 155 | [Homologous recombination](file:///D:\%E5%8D%8E%E5%A4%A7%E8%BD%AC%E5%BD%95%E7%BB%84%E6%95%B0%E6%8D%AE\GeneDiffExp\GeneDiffExp\Pathway\ACB-BtS-VS-ACB-AbR.html#gene155) | 5 (0.35%) | 110 (0.55%) | 0.8957712 | 1.000000e+00 | ko03440 |
| 156 | [Ubiquinone and other terpenoid-quinone biosynthesis](file:///D:\%E5%8D%8E%E5%A4%A7%E8%BD%AC%E5%BD%95%E7%BB%84%E6%95%B0%E6%8D%AE\GeneDiffExp\GeneDiffExp\Pathway\ACB-BtS-VS-ACB-AbR.html#gene156) | 2 (0.14%) | 53 (0.26%) | 0.8967554 | 1.000000e+00 | ko00130 |
| 157 | [Taste transduction](file:///D:\%E5%8D%8E%E5%A4%A7%E8%BD%AC%E5%BD%95%E7%BB%84%E6%95%B0%E6%8D%AE\GeneDiffExp\GeneDiffExp\Pathway\ACB-BtS-VS-ACB-AbR.html#gene157) | 1 (0.07%) | 31 (0.15%) | 0.8969803 | 1.000000e+00 | ko04742 |
| 158 | [PPAR signaling pathway](file:///D:\%E5%8D%8E%E5%A4%A7%E8%BD%AC%E5%BD%95%E7%BB%84%E6%95%B0%E6%8D%AE\GeneDiffExp\GeneDiffExp\Pathway\ACB-BtS-VS-ACB-AbR.html#gene158) | 12 (0.84%) | 231 (1.15%) | 0.8979242 | 1.000000e+00 | ko03320 |
| 159 | [Fc epsilon RI signaling pathway](file:///D:\%E5%8D%8E%E5%A4%A7%E8%BD%AC%E5%BD%95%E7%BB%84%E6%95%B0%E6%8D%AE\GeneDiffExp\GeneDiffExp\Pathway\ACB-BtS-VS-ACB-AbR.html#gene159) | 4 (0.28%) | 94 (0.47%) | 0.906273 | 1.000000e+00 | ko04664 |
| 160 | [Cytokine-cytokine receptor interaction](file:///D:\%E5%8D%8E%E5%A4%A7%E8%BD%AC%E5%BD%95%E7%BB%84%E6%95%B0%E6%8D%AE\GeneDiffExp\GeneDiffExp\Pathway\ACB-BtS-VS-ACB-AbR.html#gene160) | 6 (0.42%) | 131 (0.65%) | 0.9078595 | 1.000000e+00 | ko04060 |
| 161 | [Glutamatergic synapse](file:///D:\%E5%8D%8E%E5%A4%A7%E8%BD%AC%E5%BD%95%E7%BB%84%E6%95%B0%E6%8D%AE\GeneDiffExp\GeneDiffExp\Pathway\ACB-BtS-VS-ACB-AbR.html#gene161) | 11 (0.77%) | 218 (1.08%) | 0.9091258 | 1.000000e+00 | ko04724 |
| 162 | [Regulation of autophagy](file:///D:\%E5%8D%8E%E5%A4%A7%E8%BD%AC%E5%BD%95%E7%BB%84%E6%95%B0%E6%8D%AE\GeneDiffExp\GeneDiffExp\Pathway\ACB-BtS-VS-ACB-AbR.html#gene162) | 1 (0.07%) | 33 (0.16%) | 0.9110423 | 1.000000e+00 | ko04140 |
| 163 | [Nicotinate and nicotinamide metabolism](file:///D:\%E5%8D%8E%E5%A4%A7%E8%BD%AC%E5%BD%95%E7%BB%84%E6%95%B0%E6%8D%AE\GeneDiffExp\GeneDiffExp\Pathway\ACB-BtS-VS-ACB-AbR.html#gene163) | 3 (0.21%) | 76 (0.38%) | 0.9116479 | 1.000000e+00 | ko00760 |
| 164 | [Fc gamma R-mediated phagocytosis](file:///D:\%E5%8D%8E%E5%A4%A7%E8%BD%AC%E5%BD%95%E7%BB%84%E6%95%B0%E6%8D%AE\GeneDiffExp\GeneDiffExp\Pathway\ACB-BtS-VS-ACB-AbR.html#gene164) | 18 (1.26%) | 335 (1.66%) | 0.9119726 | 1.000000e+00 | ko04666 |
| 165 | [Glycerolipid metabolism](file:///D:\%E5%8D%8E%E5%A4%A7%E8%BD%AC%E5%BD%95%E7%BB%84%E6%95%B0%E6%8D%AE\GeneDiffExp\GeneDiffExp\Pathway\ACB-BtS-VS-ACB-AbR.html#gene165) | 17 (1.19%) | 321 (1.59%) | 0.9173203 | 1.000000e+00 | ko00561 |
| 166 | [Viral myocarditis](file:///D:\%E5%8D%8E%E5%A4%A7%E8%BD%AC%E5%BD%95%E7%BB%84%E6%95%B0%E6%8D%AE\GeneDiffExp\GeneDiffExp\Pathway\ACB-BtS-VS-ACB-AbR.html#gene166) | 9 (0.63%) | 189 (0.94%) | 0.9238246 | 1.000000e+00 | ko05416 |
| 167 | [Basal cell carcinoma](file:///D:\%E5%8D%8E%E5%A4%A7%E8%BD%AC%E5%BD%95%E7%BB%84%E6%95%B0%E6%8D%AE\GeneDiffExp\GeneDiffExp\Pathway\ACB-BtS-VS-ACB-AbR.html#gene167) | 3 (0.21%) | 79 (0.39%) | 0.924355 | 1.000000e+00 | ko05217 |
| 168 | [Shigellosis](file:///D:\%E5%8D%8E%E5%A4%A7%E8%BD%AC%E5%BD%95%E7%BB%84%E6%95%B0%E6%8D%AE\GeneDiffExp\GeneDiffExp\Pathway\ACB-BtS-VS-ACB-AbR.html#gene168) | 12 (0.84%) | 241 (1.2%) | 0.9250056 | 1.000000e+00 | ko05131 |
| 169 | [mRNA surveillance pathway](file:///D:\%E5%8D%8E%E5%A4%A7%E8%BD%AC%E5%BD%95%E7%BB%84%E6%95%B0%E6%8D%AE\GeneDiffExp\GeneDiffExp\Pathway\ACB-BtS-VS-ACB-AbR.html#gene169) | 32 (2.25%) | 569 (2.82%) | 0.9293755 | 1.000000e+00 | ko03015 |
| 170 | [Neuroactive ligand-receptor interaction](file:///D:\%E5%8D%8E%E5%A4%A7%E8%BD%AC%E5%BD%95%E7%BB%84%E6%95%B0%E6%8D%AE\GeneDiffExp\GeneDiffExp\Pathway\ACB-BtS-VS-ACB-AbR.html#gene170) | 29 (2.04%) | 523 (2.6%) | 0.9320518 | 1.000000e+00 | ko04080 |
| 171 | [Chagas disease (American trypanosomiasis)](file:///D:\%E5%8D%8E%E5%A4%A7%E8%BD%AC%E5%BD%95%E7%BB%84%E6%95%B0%E6%8D%AE\GeneDiffExp\GeneDiffExp\Pathway\ACB-BtS-VS-ACB-AbR.html#gene171) | 5 (0.35%) | 120 (0.6%) | 0.9323088 | 1.000000e+00 | ko05142 |
| 172 | [Pertussis](file:///D:\%E5%8D%8E%E5%A4%A7%E8%BD%AC%E5%BD%95%E7%BB%84%E6%95%B0%E6%8D%AE\GeneDiffExp\GeneDiffExp\Pathway\ACB-BtS-VS-ACB-AbR.html#gene172) | 5 (0.35%) | 120 (0.6%) | 0.9323088 | 1.000000e+00 | ko05133 |
| 173 | [Circadian rhythm - mammal](file:///D:\%E5%8D%8E%E5%A4%A7%E8%BD%AC%E5%BD%95%E7%BB%84%E6%95%B0%E6%8D%AE\GeneDiffExp\GeneDiffExp\Pathway\ACB-BtS-VS-ACB-AbR.html#gene173) | 1 (0.07%) | 37 (0.18%) | 0.933673 | 1.000000e+00 | ko04710 |
| 174 | [Leishmaniasis](file:///D:\%E5%8D%8E%E5%A4%A7%E8%BD%AC%E5%BD%95%E7%BB%84%E6%95%B0%E6%8D%AE\GeneDiffExp\GeneDiffExp\Pathway\ACB-BtS-VS-ACB-AbR.html#gene174) | 3 (0.21%) | 82 (0.41%) | 0.9353664 | 1.000000e+00 | ko05140 |
| 175 | [Purine metabolism](file:///D:\%E5%8D%8E%E5%A4%A7%E8%BD%AC%E5%BD%95%E7%BB%84%E6%95%B0%E6%8D%AE\GeneDiffExp\GeneDiffExp\Pathway\ACB-BtS-VS-ACB-AbR.html#gene175) | 43 (3.02%) | 747 (3.71%) | 0.9359047 | 1.000000e+00 | ko00230 |
| 176 | [Other types of O-glycan biosynthesis](file:///D:\%E5%8D%8E%E5%A4%A7%E8%BD%AC%E5%BD%95%E7%BB%84%E6%95%B0%E6%8D%AE\GeneDiffExp\GeneDiffExp\Pathway\ACB-BtS-VS-ACB-AbR.html#gene176) | 3 (0.21%) | 83 (0.41%) | 0.938695 | 1.000000e+00 | ko00514 |
| 177 | [Biosynthesis of unsaturated fatty acids](file:///D:\%E5%8D%8E%E5%A4%A7%E8%BD%AC%E5%BD%95%E7%BB%84%E6%95%B0%E6%8D%AE\GeneDiffExp\GeneDiffExp\Pathway\ACB-BtS-VS-ACB-AbR.html#gene177) | 4 (0.28%) | 103 (0.51%) | 0.9387309 | 1.000000e+00 | ko01040 |
| 178 | [Steroid hormone biosynthesis](file:///D:\%E5%8D%8E%E5%A4%A7%E8%BD%AC%E5%BD%95%E7%BB%84%E6%95%B0%E6%8D%AE\GeneDiffExp\GeneDiffExp\Pathway\ACB-BtS-VS-ACB-AbR.html#gene178) | 5 (0.35%) | 123 (0.61%) | 0.9407789 | 1.000000e+00 | ko00140 |
| 179 | [Axon guidance](file:///D:\%E5%8D%8E%E5%A4%A7%E8%BD%AC%E5%BD%95%E7%BB%84%E6%95%B0%E6%8D%AE\GeneDiffExp\GeneDiffExp\Pathway\ACB-BtS-VS-ACB-AbR.html#gene179) | 11 (0.77%) | 232 (1.15%) | 0.9426174 | 1.000000e+00 | ko04360 |
| 180 | [Thyroid cancer](file:///D:\%E5%8D%8E%E5%A4%A7%E8%BD%AC%E5%BD%95%E7%BB%84%E6%95%B0%E6%8D%AE\GeneDiffExp\GeneDiffExp\Pathway\ACB-BtS-VS-ACB-AbR.html#gene180) | 1 (0.07%) | 41 (0.2%) | 0.9505495 | 1.000000e+00 | ko05216 |
| 181 | [Neurotrophin signaling pathway](file:///D:\%E5%8D%8E%E5%A4%A7%E8%BD%AC%E5%BD%95%E7%BB%84%E6%95%B0%E6%8D%AE\GeneDiffExp\GeneDiffExp\Pathway\ACB-BtS-VS-ACB-AbR.html#gene181) | 10 (0.7%) | 220 (1.09%) | 0.952752 | 1.000000e+00 | ko04722 |
| 182 | [Dilated cardiomyopathy](file:///D:\%E5%8D%8E%E5%A4%A7%E8%BD%AC%E5%BD%95%E7%BB%84%E6%95%B0%E6%8D%AE\GeneDiffExp\GeneDiffExp\Pathway\ACB-BtS-VS-ACB-AbR.html#gene182) | 16 (1.12%) | 325 (1.61%) | 0.954374 | 1.000000e+00 | ko05414 |
| 183 | [Prostate cancer](file:///D:\%E5%8D%8E%E5%A4%A7%E8%BD%AC%E5%BD%95%E7%BB%84%E6%95%B0%E6%8D%AE\GeneDiffExp\GeneDiffExp\Pathway\ACB-BtS-VS-ACB-AbR.html#gene183) | 8 (0.56%) | 185 (0.92%) | 0.954421 | 1.000000e+00 | ko05215 |
| 184 | [Peroxisome](file:///D:\%E5%8D%8E%E5%A4%A7%E8%BD%AC%E5%BD%95%E7%BB%84%E6%95%B0%E6%8D%AE\GeneDiffExp\GeneDiffExp\Pathway\ACB-BtS-VS-ACB-AbR.html#gene184) | 14 (0.98%) | 292 (1.45%) | 0.9560566 | 1.000000e+00 | ko04146 |
| 185 | [Retinol metabolism](file:///D:\%E5%8D%8E%E5%A4%A7%E8%BD%AC%E5%BD%95%E7%BB%84%E6%95%B0%E6%8D%AE\GeneDiffExp\GeneDiffExp\Pathway\ACB-BtS-VS-ACB-AbR.html#gene185) | 7 (0.49%) | 168 (0.83%) | 0.9569399 | 1.000000e+00 | ko00830 |
| 186 | [Drug metabolism - cytochrome P450](file:///D:\%E5%8D%8E%E5%A4%A7%E8%BD%AC%E5%BD%95%E7%BB%84%E6%95%B0%E6%8D%AE\GeneDiffExp\GeneDiffExp\Pathway\ACB-BtS-VS-ACB-AbR.html#gene186) | 6 (0.42%) | 150 (0.74%) | 0.958389 | 1.000000e+00 | ko00982 |
| 187 | [GnRH signaling pathway](file:///D:\%E5%8D%8E%E5%A4%A7%E8%BD%AC%E5%BD%95%E7%BB%84%E6%95%B0%E6%8D%AE\GeneDiffExp\GeneDiffExp\Pathway\ACB-BtS-VS-ACB-AbR.html#gene187) | 7 (0.49%) | 169 (0.84%) | 0.9586838 | 1.000000e+00 | ko04912 |
| 188 | [Phenylalanine metabolism](file:///D:\%E5%8D%8E%E5%A4%A7%E8%BD%AC%E5%BD%95%E7%BB%84%E6%95%B0%E6%8D%AE\GeneDiffExp\GeneDiffExp\Pathway\ACB-BtS-VS-ACB-AbR.html#gene188) | 3 (0.21%) | 91 (0.45%) | 0.9601283 | 1.000000e+00 | ko00360 |
| 189 | [Phototransduction - fly](file:///D:\%E5%8D%8E%E5%A4%A7%E8%BD%AC%E5%BD%95%E7%BB%84%E6%95%B0%E6%8D%AE\GeneDiffExp\GeneDiffExp\Pathway\ACB-BtS-VS-ACB-AbR.html#gene189) | 4 (0.28%) | 112 (0.56%) | 0.960624 | 1.000000e+00 | ko04745 |
| 190 | [T cell receptor signaling pathway](file:///D:\%E5%8D%8E%E5%A4%A7%E8%BD%AC%E5%BD%95%E7%BB%84%E6%95%B0%E6%8D%AE\GeneDiffExp\GeneDiffExp\Pathway\ACB-BtS-VS-ACB-AbR.html#gene190) | 11 (0.77%) | 245 (1.22%) | 0.9635333 | 1.000000e+00 | ko04660 |
| 191 | [Glycosaminoglycan degradation](file:///D:\%E5%8D%8E%E5%A4%A7%E8%BD%AC%E5%BD%95%E7%BB%84%E6%95%B0%E6%8D%AE\GeneDiffExp\GeneDiffExp\Pathway\ACB-BtS-VS-ACB-AbR.html#gene191) | 1 (0.07%) | 46 (0.23%) | 0.9657442 | 1.000000e+00 | ko00531 |
| 192 | [Arrhythmogenic right ventricular cardiomyopathy (ARVC)](file:///D:\%E5%8D%8E%E5%A4%A7%E8%BD%AC%E5%BD%95%E7%BB%84%E6%95%B0%E6%8D%AE\GeneDiffExp\GeneDiffExp\Pathway\ACB-BtS-VS-ACB-AbR.html#gene192) | 6 (0.42%) | 155 (0.77%) | 0.9666154 | 1.000000e+00 | ko05412 |
| 193 | [Fat digestion and absorption](file:///D:\%E5%8D%8E%E5%A4%A7%E8%BD%AC%E5%BD%95%E7%BB%84%E6%95%B0%E6%8D%AE\GeneDiffExp\GeneDiffExp\Pathway\ACB-BtS-VS-ACB-AbR.html#gene193) | 10 (0.7%) | 231 (1.15%) | 0.9684815 | 1.000000e+00 | ko04975 |
| 194 | [MAPK signaling pathway](file:///D:\%E5%8D%8E%E5%A4%A7%E8%BD%AC%E5%BD%95%E7%BB%84%E6%95%B0%E6%8D%AE\GeneDiffExp\GeneDiffExp\Pathway\ACB-BtS-VS-ACB-AbR.html#gene194) | 21 (1.48%) | 423 (2.1%) | 0.9692478 | 1.000000e+00 | ko04010 |
| 195 | [Focal adhesion](file:///D:\%E5%8D%8E%E5%A4%A7%E8%BD%AC%E5%BD%95%E7%BB%84%E6%95%B0%E6%8D%AE\GeneDiffExp\GeneDiffExp\Pathway\ACB-BtS-VS-ACB-AbR.html#gene195) | 32 (2.25%) | 608 (3.02%) | 0.9713036 | 1.000000e+00 | ko04510 |
| 196 | [Melanogenesis](file:///D:\%E5%8D%8E%E5%A4%A7%E8%BD%AC%E5%BD%95%E7%BB%84%E6%95%B0%E6%8D%AE\GeneDiffExp\GeneDiffExp\Pathway\ACB-BtS-VS-ACB-AbR.html#gene196) | 8 (0.56%) | 199 (0.99%) | 0.974057 | 1.000000e+00 | ko04916 |
| 197 | [Calcium signaling pathway](file:///D:\%E5%8D%8E%E5%A4%A7%E8%BD%AC%E5%BD%95%E7%BB%84%E6%95%B0%E6%8D%AE\GeneDiffExp\GeneDiffExp\Pathway\ACB-BtS-VS-ACB-AbR.html#gene197) | 12 (0.84%) | 273 (1.36%) | 0.974636 | 1.000000e+00 | ko04020 |
| 198 | [N-Glycan biosynthesis](file:///D:\%E5%8D%8E%E5%A4%A7%E8%BD%AC%E5%BD%95%E7%BB%84%E6%95%B0%E6%8D%AE\GeneDiffExp\GeneDiffExp\Pathway\ACB-BtS-VS-ACB-AbR.html#gene198) | 2 (0.14%) | 79 (0.39%) | 0.9787002 | 1.000000e+00 | ko00510 |
| 199 | [Riboflavin metabolism](file:///D:\%E5%8D%8E%E5%A4%A7%E8%BD%AC%E5%BD%95%E7%BB%84%E6%95%B0%E6%8D%AE\GeneDiffExp\GeneDiffExp\Pathway\ACB-BtS-VS-ACB-AbR.html#gene199) | 1 (0.07%) | 53 (0.26%) | 0.9795141 | 1.000000e+00 | ko00740 |
| 200 | [Endometrial cancer](file:///D:\%E5%8D%8E%E5%A4%A7%E8%BD%AC%E5%BD%95%E7%BB%84%E6%95%B0%E6%8D%AE\GeneDiffExp\GeneDiffExp\Pathway\ACB-BtS-VS-ACB-AbR.html#gene200) | 3 (0.21%) | 103 (0.51%) | 0.9795263 | 1.000000e+00 | ko05213 |
| 201 | [Ascorbate and aldarate metabolism](file:///D:\%E5%8D%8E%E5%A4%A7%E8%BD%AC%E5%BD%95%E7%BB%84%E6%95%B0%E6%8D%AE\GeneDiffExp\GeneDiffExp\Pathway\ACB-BtS-VS-ACB-AbR.html#gene201) | 2 (0.14%) | 83 (0.41%) | 0.9834376 | 1.000000e+00 | ko00053 |
| 202 | [Pancreatic cancer](file:///D:\%E5%8D%8E%E5%A4%A7%E8%BD%AC%E5%BD%95%E7%BB%84%E6%95%B0%E6%8D%AE\GeneDiffExp\GeneDiffExp\Pathway\ACB-BtS-VS-ACB-AbR.html#gene202) | 3 (0.21%) | 107 (0.53%) | 0.9836858 | 1.000000e+00 | ko05212 |
| 203 | [Metabolism of xenobiotics by cytochrome P450](file:///D:\%E5%8D%8E%E5%A4%A7%E8%BD%AC%E5%BD%95%E7%BB%84%E6%95%B0%E6%8D%AE\GeneDiffExp\GeneDiffExp\Pathway\ACB-BtS-VS-ACB-AbR.html#gene203) | 5 (0.35%) | 153 (0.76%) | 0.98577 | 1.000000e+00 | ko00980 |
| 204 | [Insulin signaling pathway](file:///D:\%E5%8D%8E%E5%A4%A7%E8%BD%AC%E5%BD%95%E7%BB%84%E6%95%B0%E6%8D%AE\GeneDiffExp\GeneDiffExp\Pathway\ACB-BtS-VS-ACB-AbR.html#gene204) | 16 (1.12%) | 361 (1.79%) | 0.9857765 | 1.000000e+00 | ko04910 |
| 205 | [Serotonergic synapse](file:///D:\%E5%8D%8E%E5%A4%A7%E8%BD%AC%E5%BD%95%E7%BB%84%E6%95%B0%E6%8D%AE\GeneDiffExp\GeneDiffExp\Pathway\ACB-BtS-VS-ACB-AbR.html#gene205) | 4 (0.28%) | 134 (0.67%) | 0.9874045 | 1.000000e+00 | ko04726 |
| 206 | [Histidine metabolism](file:///D:\%E5%8D%8E%E5%A4%A7%E8%BD%AC%E5%BD%95%E7%BB%84%E6%95%B0%E6%8D%AE\GeneDiffExp\GeneDiffExp\Pathway\ACB-BtS-VS-ACB-AbR.html#gene206) | 1 (0.07%) | 60 (0.3%) | 0.9877512 | 1.000000e+00 | ko00340 |
| 207 | [Regulation of actin cytoskeleton](file:///D:\%E5%8D%8E%E5%A4%A7%E8%BD%AC%E5%BD%95%E7%BB%84%E6%95%B0%E6%8D%AE\GeneDiffExp\GeneDiffExp\Pathway\ACB-BtS-VS-ACB-AbR.html#gene207) | 29 (2.04%) | 592 (2.94%) | 0.9882536 | 1.000000e+00 | ko04810 |
| 208 | [Proximal tubule bicarbonate reclamation](file:///D:\%E5%8D%8E%E5%A4%A7%E8%BD%AC%E5%BD%95%E7%BB%84%E6%95%B0%E6%8D%AE\GeneDiffExp\GeneDiffExp\Pathway\ACB-BtS-VS-ACB-AbR.html#gene208) | 1 (0.07%) | 61 (0.3%) | 0.988619 | 1.000000e+00 | ko04964 |
| 209 | [Vascular smooth muscle contraction](file:///D:\%E5%8D%8E%E5%A4%A7%E8%BD%AC%E5%BD%95%E7%BB%84%E6%95%B0%E6%8D%AE\GeneDiffExp\GeneDiffExp\Pathway\ACB-BtS-VS-ACB-AbR.html#gene209) | 16 (1.12%) | 369 (1.83%) | 0.9892261 | 1.000000e+00 | ko04270 |
| 210 | [Transcriptional misregulation in cancer](file:///D:\%E5%8D%8E%E5%A4%A7%E8%BD%AC%E5%BD%95%E7%BB%84%E6%95%B0%E6%8D%AE\GeneDiffExp\GeneDiffExp\Pathway\ACB-BtS-VS-ACB-AbR.html#gene210) | 14 (0.98%) | 334 (1.66%) | 0.9897076 | 1.000000e+00 | ko05202 |
| 211 | [Carbohydrate digestion and absorption](file:///D:\%E5%8D%8E%E5%A4%A7%E8%BD%AC%E5%BD%95%E7%BB%84%E6%95%B0%E6%8D%AE\GeneDiffExp\GeneDiffExp\Pathway\ACB-BtS-VS-ACB-AbR.html#gene211) | 14 (0.98%) | 334 (1.66%) | 0.9897076 | 1.000000e+00 | ko04973 |
| 212 | [Aldosterone-regulated sodium reabsorption](file:///D:\%E5%8D%8E%E5%A4%A7%E8%BD%AC%E5%BD%95%E7%BB%84%E6%95%B0%E6%8D%AE\GeneDiffExp\GeneDiffExp\Pathway\ACB-BtS-VS-ACB-AbR.html#gene212) | 1 (0.07%) | 64 (0.32%) | 0.990871 | 1.000000e+00 | ko04960 |
| 213 | [Propanoate metabolism](file:///D:\%E5%8D%8E%E5%A4%A7%E8%BD%AC%E5%BD%95%E7%BB%84%E6%95%B0%E6%8D%AE\GeneDiffExp\GeneDiffExp\Pathway\ACB-BtS-VS-ACB-AbR.html#gene213) | 2 (0.14%) | 93 (0.46%) | 0.9912358 | 1.000000e+00 | ko00640 |
| 214 | [Chronic myeloid leukemia](file:///D:\%E5%8D%8E%E5%A4%A7%E8%BD%AC%E5%BD%95%E7%BB%84%E6%95%B0%E6%8D%AE\GeneDiffExp\GeneDiffExp\Pathway\ACB-BtS-VS-ACB-AbR.html#gene214) | 3 (0.21%) | 120 (0.6%) | 0.9923146 | 1.000000e+00 | ko05220 |
| 215 | [Primary immunodeficiency](file:///D:\%E5%8D%8E%E5%A4%A7%E8%BD%AC%E5%BD%95%E7%BB%84%E6%95%B0%E6%8D%AE\GeneDiffExp\GeneDiffExp\Pathway\ACB-BtS-VS-ACB-AbR.html#gene215) | 3 (0.21%) | 122 (0.61%) | 0.9931673 | 1.000000e+00 | ko05340 |
| 216 | [Cholinergic synapse](file:///D:\%E5%8D%8E%E5%A4%A7%E8%BD%AC%E5%BD%95%E7%BB%84%E6%95%B0%E6%8D%AE\GeneDiffExp\GeneDiffExp\Pathway\ACB-BtS-VS-ACB-AbR.html#gene216) | 4 (0.28%) | 146 (0.72%) | 0.9934306 | 1.000000e+00 | ko04725 |
| 217 | [Salmonella infection](file:///D:\%E5%8D%8E%E5%A4%A7%E8%BD%AC%E5%BD%95%E7%BB%84%E6%95%B0%E6%8D%AE\GeneDiffExp\GeneDiffExp\Pathway\ACB-BtS-VS-ACB-AbR.html#gene217) | 12 (0.84%) | 312 (1.55%) | 0.9943044 | 1.000000e+00 | ko05132 |
| 218 | [Arginine and proline metabolism](file:///D:\%E5%8D%8E%E5%A4%A7%E8%BD%AC%E5%BD%95%E7%BB%84%E6%95%B0%E6%8D%AE\GeneDiffExp\GeneDiffExp\Pathway\ACB-BtS-VS-ACB-AbR.html#gene218) | 6 (0.42%) | 195 (0.97%) | 0.9949874 | 1.000000e+00 | ko00330 |
| 219 | [Fanconi anemia pathway](file:///D:\%E5%8D%8E%E5%A4%A7%E8%BD%AC%E5%BD%95%E7%BB%84%E6%95%B0%E6%8D%AE\GeneDiffExp\GeneDiffExp\Pathway\ACB-BtS-VS-ACB-AbR.html#gene219) | 7 (0.49%) | 221 (1.1%) | 0.9960584 | 1.000000e+00 | ko03460 |
| 220 | [Steroid biosynthesis](file:///D:\%E5%8D%8E%E5%A4%A7%E8%BD%AC%E5%BD%95%E7%BB%84%E6%95%B0%E6%8D%AE\GeneDiffExp\GeneDiffExp\Pathway\ACB-BtS-VS-ACB-AbR.html#gene220) | 2 (0.14%) | 106 (0.53%) | 0.9962204 | 1.000000e+00 | ko00100 |
| 221 | [Gastric acid secretion](file:///D:\%E5%8D%8E%E5%A4%A7%E8%BD%AC%E5%BD%95%E7%BB%84%E6%95%B0%E6%8D%AE\GeneDiffExp\GeneDiffExp\Pathway\ACB-BtS-VS-ACB-AbR.html#gene221) | 6 (0.42%) | 204 (1.01%) | 0.9968177 | 1.000000e+00 | ko04971 |
| 222 | [Chemokine signaling pathway](file:///D:\%E5%8D%8E%E5%A4%A7%E8%BD%AC%E5%BD%95%E7%BB%84%E6%95%B0%E6%8D%AE\GeneDiffExp\GeneDiffExp\Pathway\ACB-BtS-VS-ACB-AbR.html#gene222) | 10 (0.7%) | 291 (1.44%) | 0.997356 | 1.000000e+00 | ko04062 |
| 223 | [Non-small cell lung cancer](file:///D:\%E5%8D%8E%E5%A4%A7%E8%BD%AC%E5%BD%95%E7%BB%84%E6%95%B0%E6%8D%AE\GeneDiffExp\GeneDiffExp\Pathway\ACB-BtS-VS-ACB-AbR.html#gene223) | 1 (0.07%) | 81 (0.4%) | 0.9973847 | 1.000000e+00 | ko05223 |
| 224 | [Insect hormone biosynthesis](file:///D:\%E5%8D%8E%E5%A4%A7%E8%BD%AC%E5%BD%95%E7%BB%84%E6%95%B0%E6%8D%AE\GeneDiffExp\GeneDiffExp\Pathway\ACB-BtS-VS-ACB-AbR.html#gene224) | 2 (0.14%) | 112 (0.56%) | 0.9974476 | 1.000000e+00 | ko00981 |
| 225 | [Vitamin digestion and absorption](file:///D:\%E5%8D%8E%E5%A4%A7%E8%BD%AC%E5%BD%95%E7%BB%84%E6%95%B0%E6%8D%AE\GeneDiffExp\GeneDiffExp\Pathway\ACB-BtS-VS-ACB-AbR.html#gene225) | 3 (0.21%) | 148 (0.73%) | 0.9985717 | 1.000000e+00 | ko04977 |
| 226 | [Vasopressin-regulated water reabsorption](file:///D:\%E5%8D%8E%E5%A4%A7%E8%BD%AC%E5%BD%95%E7%BB%84%E6%95%B0%E6%8D%AE\GeneDiffExp\GeneDiffExp\Pathway\ACB-BtS-VS-ACB-AbR.html#gene226) | 10 (0.7%) | 307 (1.52%) | 0.9987198 | 1.000000e+00 | ko04962 |
| 227 | [beta-Alanine metabolism](file:///D:\%E5%8D%8E%E5%A4%A7%E8%BD%AC%E5%BD%95%E7%BB%84%E6%95%B0%E6%8D%AE\GeneDiffExp\GeneDiffExp\Pathway\ACB-BtS-VS-ACB-AbR.html#gene227) | 1 (0.07%) | 96 (0.48%) | 0.9991328 | 1.000000e+00 | ko00410 |
| 228 | [Glioma](file:///D:\%E5%8D%8E%E5%A4%A7%E8%BD%AC%E5%BD%95%E7%BB%84%E6%95%B0%E6%8D%AE\GeneDiffExp\GeneDiffExp\Pathway\ACB-BtS-VS-ACB-AbR.html#gene228) | 2 (0.14%) | 129 (0.64%) | 0.9991713 | 1.000000e+00 | ko05214 |
| 229 | [Cocaine addiction](file:///D:\%E5%8D%8E%E5%A4%A7%E8%BD%AC%E5%BD%95%E7%BB%84%E6%95%B0%E6%8D%AE\GeneDiffExp\GeneDiffExp\Pathway\ACB-BtS-VS-ACB-AbR.html#gene229) | 2 (0.14%) | 137 (0.68%) | 0.9995146 | 1.000000e+00 | ko05030 |
| 230 | [Glycosaminoglycan biosynthesis - heparan sulfate](file:///D:\%E5%8D%8E%E5%A4%A7%E8%BD%AC%E5%BD%95%E7%BB%84%E6%95%B0%E6%8D%AE\GeneDiffExp\GeneDiffExp\Pathway\ACB-BtS-VS-ACB-AbR.html#gene230) | 3 (0.21%) | 166 (0.82%) | 0.9995323 | 1.000000e+00 | ko00534 |
| 231 | [Mineral absorption](file:///D:\%E5%8D%8E%E5%A4%A7%E8%BD%AC%E5%BD%95%E7%BB%84%E6%95%B0%E6%8D%AE\GeneDiffExp\GeneDiffExp\Pathway\ACB-BtS-VS-ACB-AbR.html#gene231) | 7 (0.49%) | 275 (1.37%) | 0.999749 | 1.000000e+00 | ko04978 |
| 232 | [Cell adhesion molecules (CAMs)](file:///D:\%E5%8D%8E%E5%A4%A7%E8%BD%AC%E5%BD%95%E7%BB%84%E6%95%B0%E6%8D%AE\GeneDiffExp\GeneDiffExp\Pathway\ACB-BtS-VS-ACB-AbR.html#gene232) | 4 (0.28%) | 203 (1.01%) | 0.9997557 | 1.000000e+00 | ko04514 |
| 233 | [Dorso-ventral axis formation](file:///D:\%E5%8D%8E%E5%A4%A7%E8%BD%AC%E5%BD%95%E7%BB%84%E6%95%B0%E6%8D%AE\GeneDiffExp\GeneDiffExp\Pathway\ACB-BtS-VS-ACB-AbR.html#gene233) | 2 (0.14%) | 150 (0.74%) | 0.9997978 | 1.000000e+00 | ko04320 |
| 234 | [Drug metabolism - other enzymes](file:///D:\%E5%8D%8E%E5%A4%A7%E8%BD%AC%E5%BD%95%E7%BB%84%E6%95%B0%E6%8D%AE\GeneDiffExp\GeneDiffExp\Pathway\ACB-BtS-VS-ACB-AbR.html#gene234) | 4 (0.28%) | 279 (1.39%) | 0.9999978 | 1.000000e+00 | ko00983 |
| 235 | [Bile secretion](file:///D:\%E5%8D%8E%E5%A4%A7%E8%BD%AC%E5%BD%95%E7%BB%84%E6%95%B0%E6%8D%AE\GeneDiffExp\GeneDiffExp\Pathway\ACB-BtS-VS-ACB-AbR.html#gene235) | 15 (1.05%) | 636 (3.16%) | 1 | 1.000000e+00 | ko04976 |
| 236 | [ABC transporters](file:///D:\%E5%8D%8E%E5%A4%A7%E8%BD%AC%E5%BD%95%E7%BB%84%E6%95%B0%E6%8D%AE\GeneDiffExp\GeneDiffExp\Pathway\ACB-BtS-VS-ACB-AbR.html#gene236) | 9 (0.63%) | 527 (2.62%) | 1 | 1.000000e+00 | ko02010 |
