# Supplementary material for: Transcriptome differences between Cry1Ab resistant and susceptible strains of Asian corn borer
Source: BMC Genomics. 2015 Mar 12;16(1):173. doi: 10.1186/s12864-015-1362-2 (PMC4406038; doi:10.1186/s12864-015-1362-2)
Supplement: Additional file 11: Table S10. — Primers used for q-PCR. [file 12864_2015_1362_MOESM11_ESM.docx]

Table S10 Primers used for q-PCR

| Gene ID | Primer sequence | Tm (°C) | Length of fragment (bp) |
| --- | --- | --- | --- |
| Unigene 31839 | 5'-AAGAATGTAGAAAATGGGTA-3' | 46.4 | 172 |
|  | 5'-TGTGACTTGGCTCTTTAA-3' |  |  |
| Unigene 30360 | 5'-TCCCGAAGGCAACATACT-3' | 46.7 | 157 |
|  | 5'-ATTGCGGTGCCACTTATT-3 |  |  |
| CL 3694.Contig2 | 5'-TGGGTGCGGAAGAACTAC-3' | 44.7 | 151bp |
|  | 5'-ATGGCACTATCAGACCTTTA-3' |  |  |
| Unigene 4357 | 5'-AACGAGCAGTCCATCAGA-3' | 50.8 | 149 |
|  | 5'-ACCCAGAACAGCCAAGTA-3' |  |  |
| Unigene 32178 | 5'-GATTGAGGCTAAGACAAA-3' | 45.6 | 182 |
|  | 5'-CACTAACAAATGCAGGTA-3' |  |  |
| CL 2426.Contig3 | 5'-TCTAATTTAAGATTGCGTAC-3' | 41.7 | 116 |
|  | 5'-TATGTTCTGGCCCTCATG-3' |  |  |
| Unigene 34570 | 5'-CATTTCAATCAACGACAG-3' | 44.4 | 160 |
|  | 5'-ATTTTGGAACTATACCTCA-3' |  |  |
| Unigene 32302 | 5'-GCGTTTCTATGAAGATGA-3' | 42.1 | 93 |
|  | 5'-ATTTCGATAAGTGAATGACA-3' |  |  |
| β-Actin | 5'-AACTTCCCGACGGTCAAGTCAT-3' | 60.0 | 168 |
|  | 5'-TGTTGGCGTACAAGTCCTTACG-3' |  |  |
